# Reinstating olfactory bulb-derived limbic gamma oscillations alleviates depression-like behavioral deficits in rodents

**DOI:** 10.1016/j.neuron.2023.04.013

**Published:** 2023-07-05

**Authors:** Qun Li, Yuichi Takeuchi, Jiale Wang, Levente Gellért, Livia Barcsai, Lizeth K. Pedraza, Anett J. Nagy, Gábor Kozák, Shinya Nakai, Shigeki Kato, Kazuto Kobayashi, Masahiro Ohsawa, Gyöngyi Horváth, Gabriella Kékesi, Magor L. Lőrincz, Orrin Devinsky, György Buzsáki, Antal Berényi

**Affiliations:** 1 MTA-SZTE “Momentum” Oscillatory Neuronal Networks Research Group, Department of Physiology, University of Szeged, Szeged 6720, Hungary; 2 HCEMM-SZTE Magnetotherapeutics Research Group, University of Szeged, Szeged 6720, Hungary; 3 Department of Neuropharmacology, Graduate School of Pharmaceutical Sciences, Nagoya City University, Nagoya 467-8603, Japan; 4 Department of Physiology, Osaka City University Graduate School of Medicine, Osaka 545-8585, Japan; 5 Department of Biopharmaceutical Sciences and Pharmacy, Faculty of Pharmaceutical Sciences, Hokkaido University, Sapporo 060-0812, Japan; 6 Faculty of Agriculture, University of Szeged, Szeged 6720, Hungary; 7 Neunos Inc, Boston, MA 02108, USA; 8 Department of Molecular Genetics, Institute of Biomedical Sciences, Fukushima Medical University School of Medicine, Fukushima 960-1295, Japan; 9 Department of Physiology, University of Szeged, Szeged 6720, Hungary; 10 Department of Physiology, Anatomy and Neuroscience, Faculty of Sciences University of Szeged, Szeged 6726, Hungary; 11 Neuroscience Division, Cardiff University, Museum Avenue, Cardiff CF10 3AX, UK; 12 Department of Neurology, NYU Langone Comprehensive Epilepsy Center, NYU Grossman School of Medicine, New York, NY 10016, USA; 13 Neuroscience Institute, New York University, New York, NY 10016, USA

**Keywords:** rat, depression, gamma oscillation, olfactory bulb, OBx, optogenetics, miniSOG, closed loop, electrical stimulation

## Abstract

Although the etiology of major depressive disorder remains poorly understood, reduced gamma oscillations is an emerging biomarker. Olfactory bulbectomy, an established model of depression that reduces limbic gamma oscillations, suffers from non-specific effects of structural damage. Here, we show that transient functional suppression of olfactory bulb neurons or their piriform cortex efferents decreased gamma oscillation power in limbic areas and induced depression-like behaviors in rodents. Enhancing transmission of gamma oscillations from olfactory bulb to limbic structures by closed-loop electrical neuromodulation alleviated these behaviors. By contrast, silencing gamma transmission by anti-phase closed-loop stimulation strengthened depression-like behaviors in naive animals. These induced behaviors were neutralized by ketamine treatment that restored limbic gamma power. Taken together, our results reveal a causal link between limbic gamma oscillations and depression-like behaviors in rodents. Interfering with these endogenous rhythms can affect behaviors in rodent models of depression, suggesting that restoring gamma oscillations may alleviate depressive symptoms.

## Highlights

• Suppression of OB or OB-PirC pathway decreases gamma and induces depressive symptoms
• Anti-phase closed-loop stimulation of PirC gamma resembles depressive symptoms
• Ketamine improves depressive symptoms induced by anti-phase gamma stimulation
• In-phase gamma stimulation alleviated symptoms induced by LPS

## Teaser

Li et al. report a causal link between limbic gamma oscillations and depression-like behaviors in rats. Interfering with the endogenous rhythms can affect mood-related behaviors in rat models of depression, suggesting that restoring gamma oscillations may improve depressive symptoms.

## Introduction

Major depressive disorder (MDD) is a common, severe, debilitating psychiatric illness often resistant to pharmacotherapy.^1^ The incidence and prevalence of MDD are increasing, with COVID-19 driving more than 50 million new cases.^2^ Even before COVID-19, depression was the second leading cause of global disability^2^ The efficacy of psychopharmacology is established for short term, typically 3-months, trials. Long-term follow up studies identify adverse effects including higher relapse rates with medication withdrawal versus unmedicated patients, weight gain, decreased libido, and higher suicide rates.^3^^,^^4^^,^^5^ Electroconvulsive therapy can be effective but is often complicated by long-term impairments in memory and other cognitive functions.^6^ Deep brain stimulation (DBS) and transcranial magnetic stimulation (TMS) are potential MDD therapies, but their long-term efficacy is uncertain.^7^ For drug-resistant MDD patients, alternative therapies are needed.

Coherent gamma oscillations (30–80 Hz) link brain areas by creating temporal “windows” to transfer information by enhancing excitability.^8^ Neuronal entrainment to gamma oscillations^9^ and gamma coupling between limbic areas^10^^,^^11^^,^^12^ can influence affect and emotional salience of stimuli. Neuronal network dysfunctions occur in MDD, reflected in spectral disturbances in electroencephalographic (EEG) signals.^13^ Limbic gamma power and its long-range desynchronization are potential MDD biomarkers.^14^^,^^15^ Ketamine has potent antidepressant effects in humans^16^ and animal models of depression,^17^^,^^18^^,^^19^ and increases brain-wide gamma power.^20^^,^^21^

**Figure 1:**
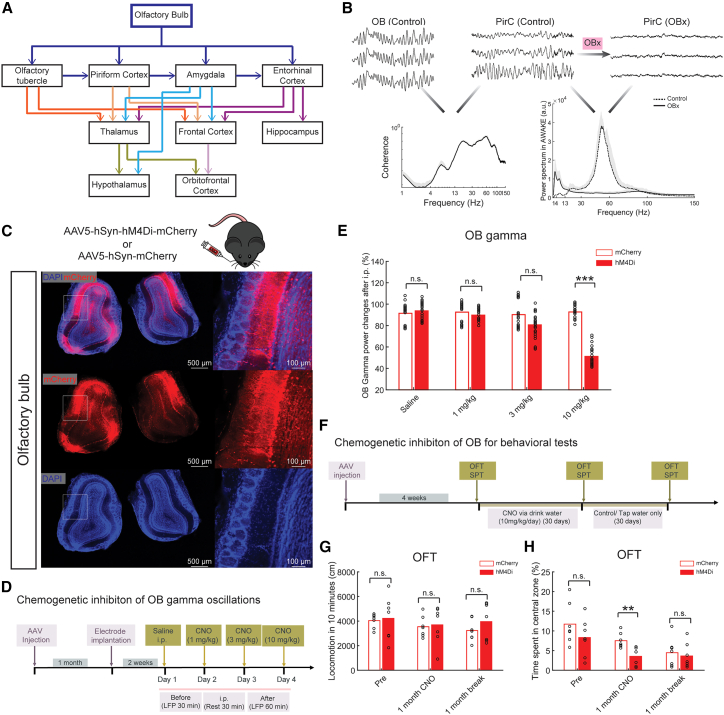
Chemogenetic inhibition of olfactory bulb neuronal activity reduces local gamma oscillations and induces depression-like behaviors (A) Major projections of the olfactory bulb (OB). (B) Top panel, represents LFPs (0.5 s) of OB and piriform cortex (PirC) from an awake rat, and LFPs in PirC after olfactory bulbectomy (OBx). Bottom panel, coherence and power spectrum corresponding to signals shown on the top panel. (C) Representative fluorescent images of the mouse olfactory bulb following injections of AAV5-hSyn-hM4Di-mCherry. (D) Schematics and timeline of the chemogenetic inhibition of OB gamma oscillations. (E) Effect of systemic administration of clozapine *N*-oxide (CNO) on OB gamma power of hM4Di and mCherry expressing mice, respectively (see Figure S2 for the same protocols carried out in the rats). (F) Schematics and timeline of the chemogenetic inhibition of OB for behavioral tests. (G) Effects of CNO on the total distance traveled in the open field test (OFT) for the hM4Di and mCherry expressing mice, respectively. The tests were performed before CNO administration (Pre), following 30 days of systemic CNO administration (1 month CNO) and following 30 days after the cessation of the CNO treatment (1 month break) (n = 7 animals/group). (H) Decreased time spent in the center zone during the OFT of the hM4Di group 1 month after CNO treatment (n = 7/group). Results of both mean value and statistical tests are reported in detail in Tables S1 and S2. n.s. indicates non-significant difference. ^∗∗^ and ^∗∗∗^ indicate differences of p < 0.01 and p < 0.001, respectively.

A physiological source of gamma oscillations is the olfactory bulb (OB, Figure 1A).^22^^,^^23^ Nasal occlusion can suppress gamma oscillations in the primary olfactory cortex (i.e., piriform cortex [PirC])^24^ and the nucleus accumbens (NAc).^25^ In rodents, bilateral olfactory bulbectomy (OBx) causes symptoms concordant with human MDD including anhedonia,^26^ failure to adapt to novel environments,^27^ increased immobility in response to stress,^28^ decreased sexual activity^29^ and learning and memory deficits.^30^ Despite lacking etiological and questioned construct validities, OBx is a frequently used animal model of depression.^31^ OBx is often criticized for the non-specific, brain wide and persistent changes in morphology and connectivity induced by the bulbectomy.^32^^,^^33^ Altered brain rhythms in the OBx model suggests however that OB-driven gamma oscillations contribute to healthy mood, although debates persists.^26^^,^^34^

To directly test this hypothesis, we combined chemogenetic, optogenetic, electrophysiological and behavioral methods with closed-loop neuromodulation of OB-derived cortical gamma oscillations in rodents. We found that reversibly suppressing the activity of OB neurons, or their efferents to PirC, suppressed limbic gamma oscillations and resembled the depression-like behaviors of OBx. Enhancing or silencing cortical gamma oscillations by the OB-driven closed-loop neuromodulation could alleviate or strengthen these depression-like behavioral deficits, respectively. The antidepressant ketamine could improve depression-like behaviors in animals with suppressed limbic gamma oscillations, suggesting that restoring gamma oscillations may improve depressive symptoms.

## Results

### Olfactory bulbectomy reduces limbic gamma oscillations and results in depression-like behaviors

Gamma oscillations in awake naive rats (Figures 1B and S1A) are highly coherent between the OB and multiple brain regions (Figures 1B and S1B–S1D). OBx dramatically reduces gamma oscillations (Figures S1E and S1F). In the PirC, the main target of OB efferents, gamma oscillations were markedly attenuated in OBx rats versus controls (Figures S1G and S1H; for descriptive statistics, tests, and sample sizes, see Table S1 and S2). Rats developed depression-like behaviors, including signs of anxiety (avoidance of open field, Figure S1I) and anhedonia (smaller sucrose water consumption, Figure S1J) 1 month following OBx, supporting that OB drives brain-wide coherent gamma oscillations that contribute to a healthy mood.

### Chemogenetic inhibition of OB neurons suppresses local gamma oscillations resulting in anxiety-like behaviors

To assess whether blocking OB-derived gamma oscillations causes depression-like symptoms, we used designer receptors exclusively activated by designer drugs (DREADDs)-based chemogenetic tools^35^^,^^36^^,^^37^ to reversibly silence pan-neuronal OB activity. We injected both OB with AAV5-hSyn-hM4Di-mCherry (Figure 1C), a modified muscarinic acetylcholine receptor selectively activated by clozapine *N*-oxide (CNO). This allowed us to suppress OB neuronal activity, thereby altering local and brain-wide gamma oscillations, without OB ablation. After systemic CNO administration, OB gamma power (30–80 Hz) was suppressed in a dose-dependent manner in mice (Figures 1D and 1E) and rats (Figure S2A). To evaluate the behavioral influence of long-term suppression of OB-induced gamma activity, mice were chronically treated with CNO (Figure 1F). The hM4Di group showed anxiety-like behavior with less time spent in the open field center (Figure 1H), but no reduction in locomotion (Figure 1G), sucrose preference test (SPT) (Figure S3A) nor daily liquid consumption (Table S3; Figures S3B and S3C). Thus, chronic OB neuronal silencing suppressed OB gamma activity and evoked anxiety-like behaviors, supporting that reduced OB gamma activity contributes to depression-like behaviors.

### Perturbing synaptic transmission from OB to PirC suppresses PirC gamma oscillations and induces anhedonia

**Figure 2:**
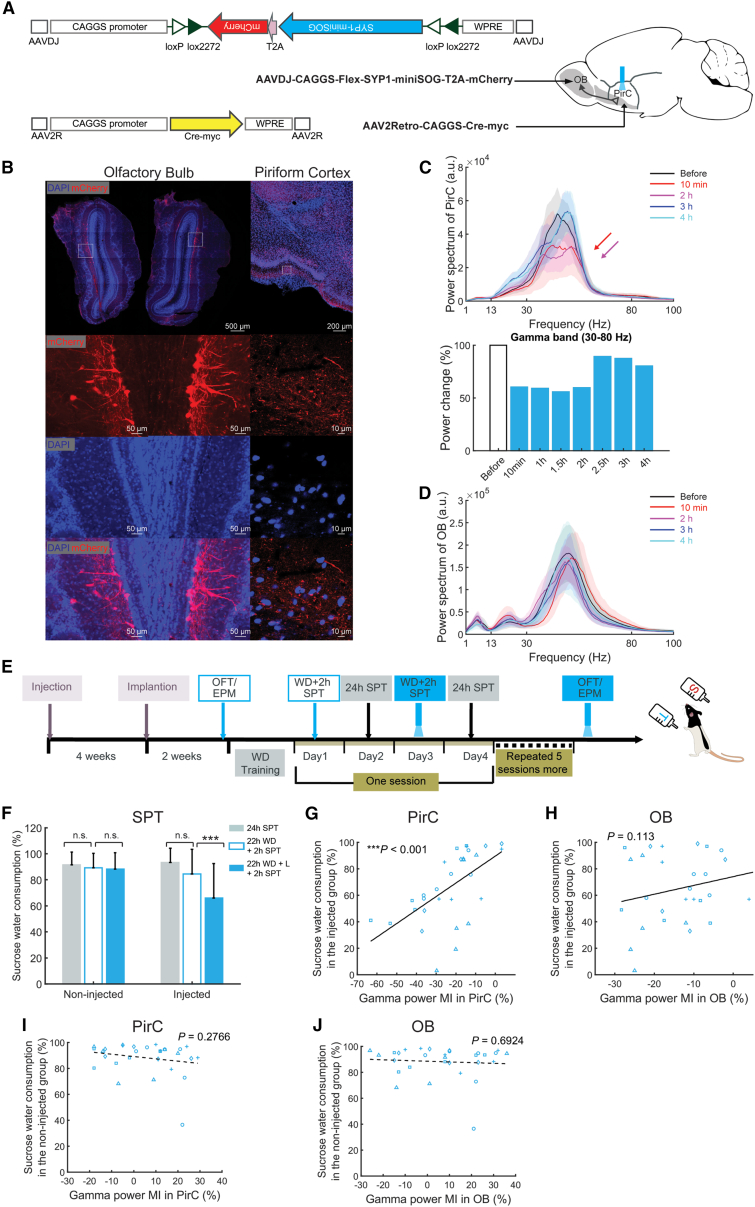
Suppressing OB to PirC synaptic transmission decreases gamma power in the PirC and deteriorates performance in the sucrose preference test (A) Schematics of the experiments and construct design for CALI (chromophore-assisted light inactivation) used for the specific inhibition of OB to the PirC synaptic transmission. (B) Fluorescent images showing mCherry expression in PirC targeting OB neurons and their axonal projections. (C) The suppression of gamma power in the PirC lasted for around 2 h after one-time illumination (450 nm light with 20 Hz for 9 min at 9 mW at the tip). Upper panels show gamma band spectrograms (30–80 Hz) before, 1 h after (1 h) and 4 h after the illumination (4 h). Bottom panel shows quantified gamma power changes in various conditions. (D) Power spectra of the OB LFPs before and during the first 4 h following illumination. (E) Schematics of the behavioral tests following the suppression of OB to PirC synaptic transmission using miniSOG. (F) Photostimulation of the PirC of miniSOG expressing rats (WD + 2 h SPT + L) decreased sucrose water consumption (120 trials from five rats/group). See performance of individual rats in Figure S5A. (G–J) Correlations between the disrupted sucrose preference performance and gamma power decrements in the PirC and the OB of the injected group (G and H) and the non-injected group (I and J) after photostimulation, respectively. Values are represented as mean ± SD. Each marker represents an individual animal. n.s. indicates not significant difference. ^∗∗∗^ indicates difference of p < 0.001. Abbreviations: OFT, open field test; EPM, elevated plus maze; WD, 22 h water deprivation; SPT, sucrose preference test; L, Illumination.

We confirmed that OB efferents to PirC projections generate and maintain coherent limbic gamma activity (Figures S1E and S1F) using pathway-specific optogenetic approaches. OB neurons projecting to PirC were selectively labeled by simultaneous viral vector injections of AAV5-EF1α-DIO-iC++-EYFP in the OB and AAV2R-CAGGS-Cre-myc in the ipsilateral PirC. This labeled all axonal arbors of ipsilateral PirC projecting OB neurons (Figure S4A). OB efferents to PirC were exclusively unilateral (Figures S4B and S4C), confirming previous reports.^38^^,^^39^ We suppressed synapses with chromophore-assisted light inactivation (CALI) to disrupt the OB to PirC projection by a single, brief light pulse, without affecting collateral paths in a spatially and temporally precise manner.^40^^,^^41^ Injection of AAVDJ-CAGGS-Flex-SYP1-miniSOG-T2A-mCherry to OB and AAV2Retro-CAGGS-Cre-myc to PirC (Figure 2A) led to viral expression exclusively in the PirC projecting OB neurons (Figure 2B).

We studied the effects of a single illumination train by recording OB and PirC local field potentials (LFPs) of awake freely moving rats (n = 5) before and after stimulation. Gamma oscillations were suppressed for 2 h following photostimulation (PS) (Figure 2C) in PirC, but not in OB (Figure 2D). We designed an SPT accounting for these temporal constrains (Figure 2E). The water-deprived animals showed reduced performance in the SPT for the duration of the suppressed gamma oscillations following the bilateral PirC PS (Figure 2F, “WD + L + 2 h”). SPT performance recovered on the subsequent day, suggesting that the anhedonic behavior did not significantly outlast the duration of the gamma suppression (Figure 2F, “24 h”). Sucrose consumption was positively correlated with gamma power in the PirC (Figure 2G), but not OB (Figure 2H). PS failed to affect these functions in naive animals (n = 5) (Figures 2F, 2I, and 2J), excluding direct non-specific effects of illumination. Similarly, the water deprivation (WD) alone did not alter the SPT performance of the injected animals (Figure 2F, “WD + 2 h”). No significant changes were found in open field test (OFT) and elevated plus maze (EPM) test following PirC PS (Figures S5B–S5E). Thus, inhibition of OB efferents to PirC for ∼2 h decreases PirC gamma oscillations resulting in anhedonia; OB-derived gamma power magnitude in the PirC predicts the magnitude of depression-like behaviors.

### Real-time silencing of OB-derived PirC gamma oscillations can induce depression-like behaviors in naive rats

**Figure 3:**
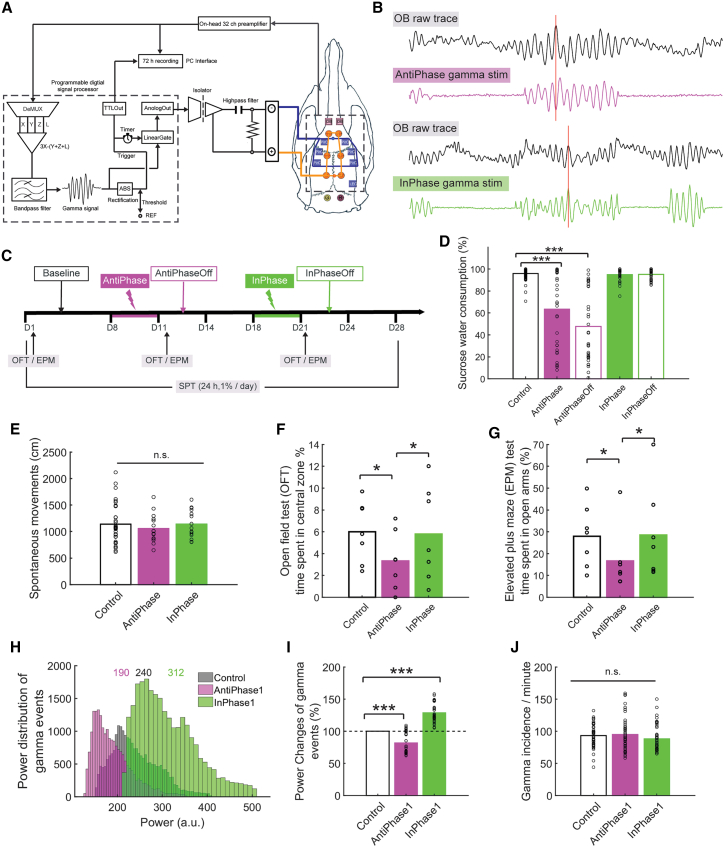
Real-time silencing of OB-derived gamma oscillations to the PirC phase-dependently induces anhedonia in naive rats (A) Schematics of closed-loop neuromodulation of the PirC with OB-derived gamma oscillations in real time. Orange circles represent miniature machine screws as cathodal (i.e., returning) electrodes. Squares indicate temporal cranial windows through which the recording and stimulating electrodes were introduced. Blue ones were for the PirC and lateral entorhinal cortex (LEC), while purple ones were for OB. Abbreviations: DeMUX, demultiplexing, artifacts were removed from the selected channel X (from OB) by subtracting averaged signals in the left (Y) and right (Z) PirC, and lateral entorhinal cortex/ventral hippocampus (L). (B) Representative LFP raw traces of OB and two derivatives for different phase stimulation (anti-phase, in-phase). Detected OB gamma oscillations were fed to the PirC with anti-phase and in-phase, respectively. Red vertical lines indicate positive peaks of original gamma oscillations in the OB. “Upward” signal represent neuronal activity (following the EEG polarity conventions) and cathodal current on the OB and stimulus traces, respectively. (C) The schema of the experiment. Each stimulation was carried out for 3 days continuously, followed by an additional 3 days without stimulation (OFF days). (D) Anti-phase stimulation significantly decreased sucrose water preference in the naive rats, and the effects lasted for 1–4 days even after termination of the stimulus. See also the individual trials in Figures S7B and S7C. In-phase stimulation did not decrease sucrose water consumption. (E) No significant side-effects of the e-stim on spontaneous movements in the homecage was observed (5 trials from four rats/group). (F and G) Anti-phase stimulation decreased the time spent in the central zone of the OFT (F) and the time spent in the open arms of the EPM test (G) in the naive rats, whereas in-phase stimulation did not alter them (n = 7 rats/group). (H) Power distribution of gamma events in the PirC on the day before the first stimulation (Base), during the first day after anti-phase stimulation ended (AntiPhase1) and during the first day after in-phase stimulation ended (InPhase1). The numbers represent medians of the distributions. See also individual five trials in the Figure S8. (I) Anti-phase stimulation significantly decreased power of gamma events in the PirC whereas in-phase stimulation increased it. (J) No significant differences of incidence of gamma events between Base, AntiPhase1 and InPhase1 in the awake state. See also time course of the gamma power changes in the PirC during in Figures S9A and S9D, gamma power changes in multiple limbic brain areas with in-phase and anti-phase stimulation in Figures S9B, S9C, S9E, and S9F and correlation map of gamma power changes in multiple limbic brain areas in Figure S9G. Each circle represents a single trial, bars indicate the population mean. n.s. indicates no significant difference. ^∗^ and ^∗∗∗^ indicate difference of p < 0.05 and p < 0.001, respectively.

Our OB-PirC gamma phase analysis revealed a coherent phase lag, indicating an inherent oscillatory entrainment through direct synaptic connections^42^ (Figure S6). We investigated whether a long-term entrainment by OB-derived PirC gamma oscillations affects depression-like behaviors. We developed an unsupervised real-time closed-loop intervention to modify PirC gamma oscillations via phase-locked (in-phase, anti-phase) electrical stimulation (e-stim) driven by OB gamma oscillations (Figures 3A–3C). Anti-phase gamma e-stim (i.e., interfering with PirC rhythmic neuronal activity) decreased sucrose preference during and after stimulation (Figures 3D and S7). In contrast, in-phase e-stim had no effect on sucrose preference (Figure 3D). Neither in-phase nor anti-phase e-stim affected the rats’ spontaneous homecage locomotion (Figure 3E). In naive rats, anti-phase e-stim induced depression-like symptoms (i.e., decreased time spent in central zone of OFT [Figure 3F] and in open arms of EPM test [Figure 3G]). In-phase e-stim had no effect, confirming the specificity of anti-phase e-stim. In-phase e-stim did not boost performance of naive, healthy rats. LFP analysis showed that anti-phase e-stim decreased gamma power in PirC while in-phase e-stim increased gamma power. These changes persisted for 1 day after stimulations (Figures 3H, 3I, and S8A). Neither in-phase nor anti-phase e-stim changed gamma frequency distribution in the PirC (Figure S8B) and gamma events incidence during wakefulness was unaltered (Figure 3J; see Figure S9 for gamma power time course in PirC and other areas). Therefore, closed-loop OB gamma neuromodulation of PirC enhances and silences PirC gamma oscillations in a phase-dependent manner, with effects persisting at least for 1 day after stimulation. The anti-phase gamma stimulation and resulting decrease in PirC gamma oscillations caused anhedonia and anxiety, consistent with a depression-like state.

### Ketamine alleviates depression-like behaviors by increasing gamma oscillations in rats with suppressed PirC gamma oscillations

**Figure 4:**
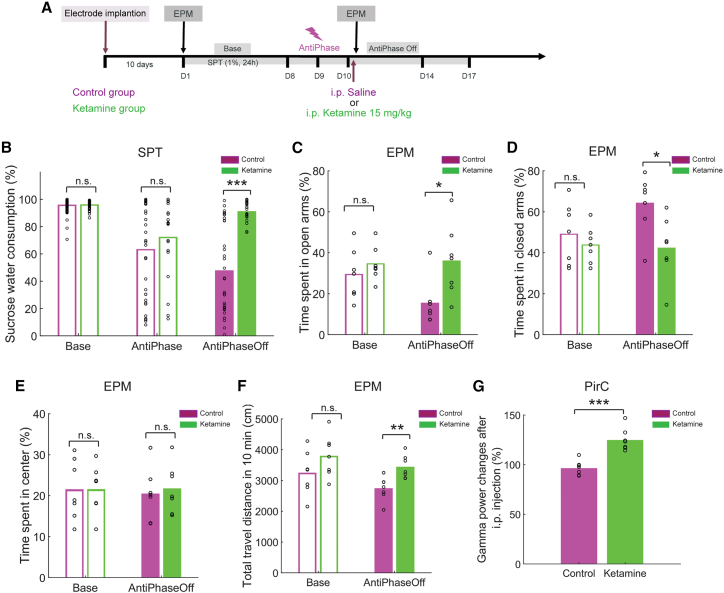
Ketamine alleviates depression-like behaviors by restoring limbic gamma oscillations (A) Schematics of the experiment. (B) Ketamine improves the decreased sucrose preference following closed-loop anti-phase PirC e-stim (n = 7 rats/group). (C–F) Ketamine alleviates the anxiety-like behaviors in the EPM test induced by the closed-loop anti-phase PirC e-stim. (G) Increased PirC gamma power following ketamine administration. For gamma power changes in various other limbic brain areas following ketamine administration see Figure S10. Circles indicate individual trials. n.s. indicates not significant difference. ^∗^, ^∗∗^, and ^∗∗∗^ indicate difference of p < 0.05, p < 0.01, and p < 0.001, respectively.

To assess whether antidepressants improve the anti-phase closed-loop PirC e-stim induced depression-like symptoms, rats were treated with ketamine 3 days following anti-phase e-stim (Figure 4A). Similar to the previous experiment, anti-phase e-stim induced anhedonia in the SPT lasting several days following stimulation in control animals (Figure 4B). Ketamine treatment improved SPT performance (Figure 4B). Anti-phase e-stim also induced anxiety-like behaviors in the EPM test in control, but not in ketamine-treated animals (Figures 4C and 4D). Ketamine increased gamma power in PirC (Figure 4G) and other limbic brain areas (Figure S10). Thus, ketamine can restore limbic gamma oscillations and revert the depression-like behaviors induced by suppression of OB-derived PirC gamma oscillations in rats.

### Real-time enhancing of OB-derived PirC gamma oscillations alleviates depression-like behaviors

**Figure 5:**
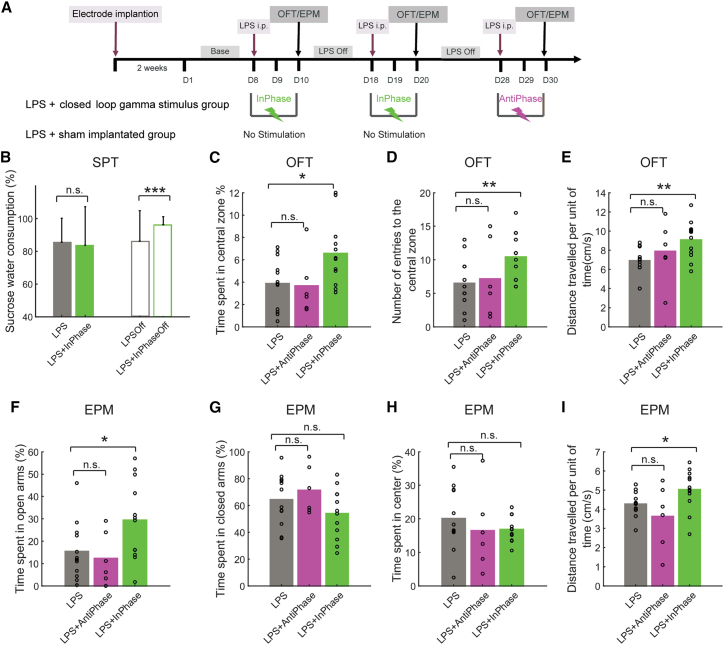
Reinstating OB-derived gamma oscillations to the PirC phase-dependently alleviates depression-like behaviors (A) Schematics of the experiment. (B) LPS decreased the sucrose preference in both groups by systemic administration of lipopolysaccharide (LPS). In-phase stimulation recovered the decreased SPT performance in the post stimulation days. See also performances of individual rats during the two in-phase stimulation sessions in Figure S11 (n = 6 rats/group). (C–I) In-phase stimulation alleviated the depression-like behaviors in the OFT (C–E) and the anxiety-like behaviors in the EPM test (F–I) induced by the LPS administration. Note that anti-phase stimulation failed to reproduce these behavioral benefits. Values are presented as mean ± SD. Circles indicate individual trials. n.s. indicates not significant difference. ^∗^, ^∗∗^, and ^∗∗∗^ indicate difference of p < 0.05, p < 0.01, and p < 0.001, respectively.

To investigate whether boosting PirC gamma oscillations by electrical stimulation can alleviate depression-like behaviors, we used in-phase closed-loop PirC e-stim in the lipopolysaccharide (LPS) model of depression in rats^43^ and mice^44^ (Figure 5A). In the SPT, LPS decreased sucrose preference, but the group receiving in-phase gamma e-stim recovered SPT performance (Figures 5B and S11). in-phase e-stim also increased the “center time” during the OFT (Figure 5C), number of center entries (Figure 5D) and distance traveled per time unit (Figure 5E) compared to non-stimulated animals. Similarly, in-phase gamma e-stim alleviated anxiety-like behaviors in the EPM test (Figure 5F), and increased distance traveled per time unit (Figure 5I), but did not alter time in closed arms (Figure 5G) or in center (Figure 5H). In rats, anti-phase e-stim did not improve behavior in OFT (Figures 5C–5E) and EPM tests (Figures 5F–5I). Thus, OB-derived phase-matched closed-loop gamma neuromodulation in PirC alleviated depression-like and anxiety-like behaviors in the SPT, OFT and EPM tests, standard rodent models of depression.

## Discussion

Our experiments causally link deficient gamma activity and behavioral impairments concordant with symptoms of MDD.^45^ The brain-wide suppression of gamma oscillations in OBx animals parallel gamma oscillopathies in MDD patients.^14^^,^^46^ Further research should assess the reciprocal link between altered OB-derived gamma oscillations in PirC and behavioral changes. Our findings, along with those of previous studies, suggest that PirC plays a significant role in the network interactions underlying depression.^47^^,^^48^^,^^49^ PirC has widespread limbic connections^50^^,^^51^ that modulate emotional states.^52^^,^^53^^,^^54^ Manipulations within one node can alter activity across brain regions and influence emotional behavior.^49^^,^^55^^,^^56^ Therefore, gamma oscillations originating from the OB may alter emotional behavior by influencing activity in PirC and its limbic connections. Gamma oscillations organize dynamics within cortical networks^8^^,^^57^ and reactivate limbic neuronal ensembles that are primed during positive experience and can ameliorate depression-like behaviors.^58^ PirC neurons respond to reward and noxious stimuli,^59^ and encode multimodal, hedonic, and context-dependent representations.^60^ PirC stimulation is self-reinforcing, likely via the induction of an internally rewarding state.^61^

While the link between gamma oscillations and depression-like behavioral deficits is strong in several animal models,^14^ the evidence linking gamma oscillations to depression in humans has been less conclusive. However, novel evidence supports gamma oscillations as a MDD biomarker^62^ and gamma oscillation based closed-loop neuromodulation can alleviate depressive symptoms in humans.^15^

Some behaviors were affected differently by pan-neuronal OB silencing versus modulation of ongoing gamma oscillations. For example, chemogenic silencing of OB neurons altered anxiety-related behaviors, but not hedonic behaviors, which were more strongly influenced by selectively manipulating OB-derived gamma oscillations. However, as a potential explanation, the former might also reflect fluctuating plasma CNO levels during behavioral tasks. Pan-neuronal silencing of OB-induced broad changes across multiple frequencies, which may underlie the observed differences in behavioral effects. Boosting gamma oscillations led to positive changes in animals with depression-like behaviors, but not in naive animals. This suggests either a specific effect to restore normal functions or a ceiling effect. Our results suggest that components of the complex repertoire characterizing depression-like behaviors in rodents may depend on various functions of both the OB and limbic system; from global neuronal activity to phase specific entrainment of neuronal ensembles.

While olfaction is a dominant sensory modality in rodents, while it is not true for humans. However, human olfaction and depression are linked. Depression is associated with reduced OB volume.^63^^,^^64^^,^^65^ Depressed patients have altered odor thresholds,^66^^,^^67^^,^^68^ impaired odor identification^69^^,^^70^ and discrimination, respectively.^65^^,^^69^ Approximatively one-third of patients with anosmia develop depressive symptoms.^71^^,^^72^ Finally, boosting olfactory function with olfactory training ameliorates depression.^73^

We do not claim that the olfactory sensory deficits are a major driver of MDD. The pathophysiology of this disorder remains poorly defined. We found that depression-like symptoms are bidirectionally affected by interfering with gamma oscillations in the rodent olfactory system (i.e., alleviating them with constructive and/or exacerbating them with destructive interference). This is consistent with evidence from human studies demonstrating that the power of gamma oscillations in the amygdala are a robust biomarker for MDD^62^ and modulating gamma activity can ameliorate depressive symptoms in patients.^15^ We conclude that controlling gamma oscillations at their source offers therapeutic potential by targeting a choke point to relieve depressive pathophysiology and symptoms.

## STAR★Methods

### Key resources table

**Table:** 

REAGENT or RESOURCE	SOURCE	IDENTIFIER
**Bacterial and Virus Strains**		

AAV5-hSyn-mCherry	Addgene	Cat# 114472-AAV5
AAV5-hSyn-hM4Di-mCherry	Addgene	Cat# 50475-AAV5
pAAV-hSyn-mCherry	Addgene	Cat# 114472
pAAV-hSyn-hM4Di-mCherry	Addgene	Cat# 50475
pAAV-SYP1-miniSOG-T2A-mCherry	Addgene	Cat# 50972
AAVDJ-CAGGS-Flex-SYP1-miniSOG-T2A-mCherry	This paper	N/A
AAV2R-CAGGS-Cre-myc	This paper	N/A
AAV5-EF1α-DIO-iC++-EYFP	UNC Vector Core	N/A

**Chemicals, Peptides, and Recombinant Proteins**		

Clozapine N-oxide	Sigma-Aldrich	Cat# C0832
Lipopolysaccharides from Escherichia coli O111:B4	Sigma-Aldrich	Cat# L2630
Urethane	Sigma-Aldrich	Cat# U2500
Paraformaldehyde	Sigma-Aldrich	Cat# P6148
DAPI	Sigma-Aldrich	Cat# D8417
Ketamine	Gedeon Richter Plc., Hungary	Calypsol

**Software and Algorithms**		

MATLAB R2017b	Mathworks	RRID:SCR_001622
Neuroscope	Hazan et al.^74^	RRID:SCR_002455
EthoVision XT software	Noldus	RRID:SCR_000441
ZEN Digital Imaging for Light Microscopy	Carl Zeiss	RRID:SCR_013672
Circular Statistics Toolbox	Berens^75^	RRID:SCR_016651
Chronux Toolbox	Bokil et al.^76^	RRID:SCR_005547
GammaDetection	This paper	https://doi.org/10.5281/zenodo.7814287

**Other**		

HML Insulated Tungsten 99.95% wire	California Fine Wire	Cat# CFW2044436

### Resource availability

#### Lead contact

Further information and requests for resources should be directed to and will be fulfilled by the lead contact, Antal Berényi (drberenyi@gmail.com).

#### Materials availability

All Virus used in this study are available from the lead contact. A completed materials transfer agreement may be needed in some cases.

### Experimental model and subject details

#### Animals and ethical approval

In total 20 adult male wild-type C57BL/6 mice (3 months old, 20–30 g) and 57 wild-type male Long-Evens rats (3–4 months old, 300–400 g) were used in this study. Both mice and rats were provided with a commercial diet and water *ad libitum* under a 12 h light/dark environment (light onset at 7 A.M.). The animals were housed as groups (3 animals/cage) before surgery, and then individually for the duration of the whole experiment. All animal studies and experimental procedures were approved by the Ethical Committee for Animal Research at the Albert Szent-Györgyi Medical and Pharmaceutical Center of the University of Szeged (XIV/218/2016, XIV/1248/2018 and XIV/824/2021) and conformed to European Union guidelines (2003/65/CE) and the National Institutes of Health Guidelines for the Care and Use of Animals for Experimental Procedures.

#### Surgical interventions

Procedures of animal surgery were discussed in detail earlier.^77^ Briefly, animals were anesthetized with 1%–3% isoflurane and then mounted on a stereotaxic apparatus (David Kopf instruments), where the anesthesia was maintained (1-2% Isoflurane). Atropine (0.1 mg/kg, s.c.) was administered immediately after the anesthesia induction. Stages of anesthesia were maintained by confirming the lack of nociceptive reflex. The rectal temperature was maintained at 36–37°C with a DC temperature controller (TMP-5b; Supertech, Pécs, Hungary). The head position was adjusted so that the bregma and lambda were at the same level. Subcutaneous lidocaine injection was administered before skin incisions. Experiment specific interventions were performed in aseptic conditions as detailed in the subsequent sections. Wounds were closed and treated by standard surgical methods. Animals were recovering for at least 3 days with appropriate antibiotic and analgetic supplement.

#### Olfactory bulbectomy experiments

Anaesthetized animals were mounted in a stereotaxic apparatus, the skull exposed, and two holes were drilled at coordinates (AP: –7, ML: ± 2, all mm from the bregma). Bilateral olfactory bulbs were removed by suction, then holes filled with haemostatic sponge. Control animals underwent similar surgical procedures except OB removal. The behavioral tests were performed one month following recovery (Figures S1I and S1J).

#### Chronic implantation of recording electrodes

Tripolar tungsten electrodes for intracranial recording were prepared as previous described.^78^ For the purpose of exploring the relationship between the olfactory bulb and putatively relevant brain areas in intact animals (Figures S1A–S1D), electrodes were implanted in two naïve rats into the following brain areas (see also Table S4): OB (olfactory bulb), PrL/IL (prelimbic cortex/ infralimbic cortex), NAc (nucleus accumbens), PirC (piriform cortex), vHip (ventral hippocampus), CeA/BLA (central amygdala/basolateral amygdala), VTA (ventral tegmental area). To investigate LFPs in the OBx animals (Figures S1E–S1H), three animal per group were implanted at 30 recording sites after the behavioral tests. Electrodes were distributed to the right hemisphere with brain areas as follows: PrL/IL, M2 (secondary motor cortex), mid NAc (medial nucleus accumbens), lat NAc (lateral nucleus accumbens), PirC, ant CgC (anterior cingulate cortex), post CgC (posterior cingulate cortex), S1 (primary somatosensory cortex), EP (entopeduncular nucleus), and VPL (ventral posterolateral thalamic nucleus). Recordings commenced after a recovery period of at least 14 days following surgery in the present study.

#### Chemogenetic inhibition of OB neurons

For exploring gamma power changes following OB chemogenetic silencing, six mice: AAV5-hSyn-mCherry (n = 3, control), AAV5-hSyn-hM4Di-mCherry (n = 3, treated), total injection sites: 18/animal, 0.2 μl/site; and six rats: AAV5-hSyn-mCherry (n = 2, control), AAV5-hSyn-hM4Di-mCherry (n = 4, treated), total injection sites: 30/animal, 0.3 μl/site were injected (injection coordinates are shown in Table S5). One month later, all animals were bilaterally implanted with LFP recording electrodes into the OB and PirC (coordinates are shown in Table S4). Two weeks following electrode implantation, saline, 1 mg/kg CNO, 3 mg/kg CNO and 10 mg/kg CNO were systemically administrated in each animal during four consecutive days (Figure 1D). Each session started with a 30 min baseline recording followed by the intraperitoneal injection of CNO/saline (under isoflurane anesthesia maintained for 5 minutes to minimize stress followed by 30 minutes of rest to allow diffusion) and 60 minutes of test recording. For the long-term modulation of OB gamma oscillations through hM4Di receptors, 14 mice were injected with either AAV5-hSyn-mCherry or AAV5-hSyn-hM4Di-mCherry. In total 18 injection sites (0.2 μl/injection site) were distributed across the OB (for details see Table S5). Following four weeks of recovery the mice received 5 ml of CNO solution (10 mg/kg/day) for 30 days supplemented in their drinking water. The solutions were freshly prepared every day and placed in aluminium foil covered bottles to avoid photolytic effects on CNO stability. Behavioral tests were evaluated three times for each mouse: before CNO treatment, after one-month of CNO treatment and after 1 month break following CNO treatment as shown in Figure 1F. Daily consumptions of CNO are shown in Table S3.

#### Optogenetic inhibition of the OB to PirC synaptic transmission

##### Preparation of a retrograde Cre-expressing AAV vector

AAV vector serotype 2-R (retrograde) was prepared based on AAV Helper-Free system (Agilent Technologies), as described in Kato et al.^79^ The transfer plasmid contained the cDNA encoding Cre recombinase and myc tag sequences downstream of the CAGGS promoter. HEK293T cells were transfected with the transfer, an adeno-helper, and expressing the adenoviral genes required for AAV replication and encapsidation plasmid through the calcium phosphate precipitation method. The crude viral lysate was purified with two rounds of CsCl gradient centrifugation, dialyzed, and concentrated with an Amicon filter (Merck Millipore). The viral genome titer was determined by quantitative PCR.

##### Preparation of an anterograde Cre-dependent miniSOG-expressing AAV vector

The core concept of the “mini–Singlet Oxygen Generator” (miniSOG) intervention is based on the optogenetic inhibition of synaptic release with chromophore-assisted light inactivation technique, which is realized by a miniSOG enzyme fused to the C terminus of the synaptophysin 1 synaptic vesicle protein (SYP1). After light illumination, singlet oxygen is generated by miniSOG leading to the inactivation of fusion protein^40^ AAV vector serotype DJ was prepared based on AAV Helper-Free system (VPK-400-DJ, Cell Biolab). The transfer plasmid contained the cDNA encoding a FLEXed InSynC (SYP1-miniSOG-T2A-mCherry) sequence downstream of the CAGGS promoter 7. HEK293T cells were transfected with the transfer, an adeno-helper, and expressing the adenoviral genes required for AAV replication and encapsidation plasmid through the polyethylenimimine method. The crude viral lysate was purified using discontinuous iodixanol gradients.^80^ The viral genome titer was determined by quantitative PCR.

##### Construction of optoprobes

Optical fiber was made as previously reported^81^ A 0.39 NA, Ø200 μm core multimode optical fibers (FT200EMT, Thorlabs) were assembled with a stainless-steel ferrule terminals (SF230, Thorlabs) and then polished on one side; exposed the silica core on 1 cm, removed TECS cladding and shaped with hydrofluoric acid on the another side. Only optoprobes with ∼9 mW maximal current output power at the tip were used, as confirmed by a photodiode power sensor (S130C, Thorlabs) and a power meter (PM200, Thorlabs). After that, each optical fiber was glued to a single tungsten wire by a UV-curing optical adhesive (NOA61, Thorlabs), the tip of wire was 0.5 mm longer than the tip of optical fiber.

##### Behavioral and electrophysiological tests

Five adult male rats were bilaterally injected with AAVDJ-CAGGS-Flex-SYP1-miniSOG-T2A-mCherry (30 sites across the OB) and AAV2R-CAGGS-Cre-myc (6 sites across the PirC), injection coordinates are shown in Table S5. Four weeks later, each rat was implanted with two tripolar electrodes in the bilateral OB and two optoprobes in the bilateral PirC. After two weeks of recovery animals underwent water deprivation (WD) training as follows. Protocol consisted of alternating days of i) 24 h SPT with ad libitum access to both water and sucrose solution and ii) 22 h of water deprivation followed by the SPT for 2 h. The WD training was lasting around two weeks until all animals reached ∼ 90% sucrose consumption during the 2 h SPT following WD. During the formal experiment (Figure 2E), each session (lasting four days) contained the following blocks: i) 2 h SPT following 22 h WD; ii) 24 h SPT without WD; iii) 2 h SPT following 22 h WD, preceded by photostimulation (a 9 min long 20 Hz train of 450 nm 25 ms light pulses, 9 mW at both fiber tips in bilateral PirC); and iv) 24 h SPT without WD. To avoid developing a place preference the location of the tap water bottle and sucrose water bottle were randomly changed each day. These four-day long sessions were repeated six times. Open field test (OFT) and elevated plus maze (EPM) test were implemented before WD training and also after the sixth session. The second OFT and EPM tests were performed following the same photostimulation protocol as the one during the six test sessions previously described. For all photostimulation experiments, 10 min LFP was recorded from the PirC before and after light delivery. The control group (n = five rats) followed identical procedure as above but without the virus injection to investigate the side effects of photostimulation (Figure 2E).

#### Closed-loop OB derived gamma neuromodulation of PirC

##### Electrode implantation surgery

Each animal was chronically implanted with intracranial recording and stimulating electrodes in the following areas: two tripolar electrodes in bilateral OB for recording gamma oscillations for online detection, one tripolar electrode in LEC for removing global EMG noise during online gamma detection, six bipolar, combined recording and stimulating electrodes in six bilateral locations of the PirC for injecting gamma activities which were detected from the OB under selective modulating parameters (in-phase and anti-phase). Bipolar stimulation electrodes in the PirC were prepared as follows, two tungsten wires axially spaced 0.3 mm apart, the tip of deep one was stripped around 0.2–0.3 mm for electrical stimulation. Recording electrodes were connected to a signal multiplexing headstage (HS3_v1.3, Amplipex, Szeged, Hungary) through short wires for long-term freely-moving recordings’ purpose. During the chronic implantation, all six stimulus wires from six locations of PirC were connected together to one pin of connector as anode current input, and six stainless-steel machine screws were installed in the skull and then combined to the other pin of the connector as cathode (Figure 3A). The connector was glued to the edge of the copper mesh with dental cement (Unifast Trad, GC). Two miniature machine screws were installed above the cerebellum as reference and ground, respectively. The stereotaxic coordinates are shown in Table S4. To investigate gamma oscillation changes in limbic brain areas, seven additional rats were implanted with electrodes in six additional brain areas besides the OB and PirC (i.e. “eight-region model”). The stereotaxic coordinates (n = 7 rats) of the additional electrode coordinates are shown in Table S4.

##### Long-term closed-loop OB derived gamma neuromodulation in freely-moving animals

Each stimulation block lasted for three continuous days performed in the home cage of the animals starting at 7 P.M. For achieving undisrupted online stimulation, care was taken to avoid twisting and over-tension of the cables as previously described.^82^ Briefly, a thin and light recording cable (40 AWG Nylon Kerrigan-Lewis Litz wire, Alpha Wire, Elizabeth, NJ, USA) was connected to a suspended commutator (Adafruit, New York, NY, USA) sliding vertically on guide rails to avoid the twisting and over-tension of the cables. The continuously recorded LFP signals in the OB were used to feed the endogenous gamma band oscillations by real-time closed-loop electrical stimulation to multiple sites in the PirC. The pre-amplified and multiplexed analogue LFP signals were fed to a programmable digital signal processor (RX-8, Tucker-Davis Technologies, Alachua, FL, USA) and to the data acquisition system (KJE-1001, Amplipex), both sampled at 16 kHz. The signals were demultiplexed and the OB channels were analysed online to detect gamma events using a custom-made signal detection algorithm based on a previously established routine.^82^ Briefly, LFP signals were demultiplexed at 500 Hz per channel and a signal from a pre-selected OB channel was band-pass filtered with a 4th order Butterworth filter to 30–110 Hz. Common artefacts were removed from the selected channel by subtracting averaged signals in the left and right PirC, and LEC/vHip. Electrical stimulation (maximum duration: 300 ms) was triggered when a filtered artefact attenuated OB signal exceeded a fine-tuned adaptive threshold for each animal. Time resolution of the detection was 2 ms. The same filtered OB gamma signals was used as electrical stimulation (e-stim) and when a gamma detection occurred, it was fed to the six PirC locations via an analog isolator IC (ISO124, Texas Instruments) through a RC high-pass filter of 0.25 s time constant (Figure 3A). For in-phase stimulation, the filtered and gated gamma waveforms were fed without inversion. For anti-phase stimulation, the signals were inverted (Figure 3B).

##### Experimental procedures for naïve animals

Two weeks after implantation, 24 h SPT was recorded every day until the experiment was completed (Figures 3C and 3D). SPT, OFT and EPM tests were performed. In total, 11 rats were exposed to the closed-loop gamma stimulation, four of them receiving both anti-phase and in-phase stimulations according to the procedure shown in Figures 3C and S7B (Baseline (7 days) – anti-phase (3 days) – Off (7 days) – in-phase (3 days) – Off (7 days)). Spontaneous movements of these four rats in the homecages were captured by a camera from 7–9 p.m. for one week as baseline, and on each stimulation days (Figure 3E). LFP recordings were collected between 7–9 p.m. before stimulation as baseline and on the day after each e-stim session (InPhase1/AntiPhase1, Figures 3H–3J). To confirm the lack of eventual accumulating e-stim effects from one stimulation block to another, six of the rats was also exposed to an opposite order of phase sequences as follows: Baseline (7 days) – in-phase (3 days) – Off (7 days) – anti-phase (3 days) – Off (7 days) (Figure S7C), and were also tested in OFT and EPM tests immediately after each stimulation phase (Figures 3F and 3G). The first three days of each seven day Off periods following the stimulation blocks were investigated to reveal any lasting effects of gamma e-stim. To investigate the time course of the stimulation induced gamma power changes in various brain areas, 30 minutes of LFP recording were collected every day through the whole procedure in seven rats with electrodes in multiple brain areas (Figure S9).

##### Experimental procedures for ketamine treatment

Ketamine (Calypsol, Gedeon Richter Plc., Hungary) was dissolved in sterile 0.9% saline and injected i.p. into the treatment group using a dose of 15 mg/kg following three days anti-phase gamma stimulation. The control group received the same volume of saline. Both groups performed the EPM test one hour following the injection. Thirty minutes of LFP recordings were recorded before i.p. injection and after the behavioral test, respectively (Figure 4A) to quantify the changes in gamma oscillations.

##### Experimental procedures for depression model animals

Lipopolysaccharide (LPS, O111:B4, Sigma) was dissolved in sterile 0.9% saline and injected i.p. into both of groups using a dose of 200 μg/kg. Animals from the implanted group were immediately connected to recording/stimulating apparatus for real time in-phase gamma stimulus from 7 p.m. until the third day morning. The sham group received no stimulation. On the third day, both groups were tested for OFT and EPM behaviors (Figure 5A).

#### Behavioral tests

##### Sucrose preference test

The sucrose preference test was performed using a two-bottle procedure, during which animals had free access to both water and a sucrose solution. Individually housed animals were presented after habituation with two bottles one with tap water and one with a 1% sucrose solution. The consumption of water and sucrose solutions was measured by weighing the bottles before and after the tests. The position of the bottles (left or right) was alternated between each test. The ratio of sucrose solution relative to the total intake (water + sucrose solution) during 24 h is considered as the sucrose consumption (%).

##### Open field test

Animals were tested for 10 mins in an open-field arena (100×100 cm for rats, 50×50 cm for mice) in dim light (75 lx) following 1h habituation in the room. Distance travelled, time spent in the center area and number of entries into the center were quantified by EthoVision XT software (Noldus, Wageningen, The Netherlands).

##### Elevated plus maze test

The maze was made of wooden board with a light grey painted matte floor, and consisted of two open arms and two closed arms (40 cm high walls with black wallpaper). Each arm was 50 cm long and 10 cm wide. The same camera and light intensity as in the OFT were used here. Rats were placed into center headed to the open arms, and video monitored for 10 mins. The time spent in the open arms, closed arms, center area and the total distance travelled during the 10 mins were quantified by EthoVision XT software.

##### Spontaneous movements

In the closed-loop gamma e-stim experiments, the spontaneous movements of rats were continuously monitored by a camera mounted above the home cage from 7 to 9 pm during the SPT experiments.

#### Histology

For verifying the virus vector-mediated gene transduction and recording electrode placements, the animals were deeply anesthetized (1.5 g/kg urethane (i.p.)) and transcardially perfused with physiological saline (0.9% NaCl) followed by 4% paraformaldehyde (PFA) solution. For the implanted animals, one recording site in each brain area was lesioned with anodal direct current for 10 s before perfusion (Rat, 100 μA; mouse, 40 μA) (Figure S7A). Brains were post-fixed overnight in 4% PFA, sectioned to 50 μm thick slices using a vibrating microtome (VT1000S, Leica, Buffalo Grove, IL, USA). The slices were then stained with 1 μg/ml 4′, 6-Diamidino-2-phenylindole dihydrochloride (D8417; Sigma-Aldrich, St. Louis, MO, USA) in distilled water. Fluorescent signals were examined with a Zeiss LSM880 laser scanning confocal microscopy (Carl Zeiss, Oberkochen, Germany). Images were acquired using a Plan-Apochromat 20×/0.8 M27 or an alpha Plan-Apochromat 63×/1.46 Oil Korr M27 objective lens. All data analysis and statistical analysis were performed in MATLAB (RRID: SCR_001622; Mathworks, Natick, MA, USA).

### Quantification and statistical analysis

All LFP data and statistical analysis were performed in MATLAB (RRID: SCR_001622; Mathworks, Natick, MA, USA). Behavioral analysis was performed using EthoVision XT software (RRID: SCR_000441, Noldus), unless otherwise stated.

#### Power spectrum and coherence analysis

Signals were pre-processed with down sampling to 1250 Hz if sampling rate was original 20 kHz. Power spectra were calculated in MATLAB using Multitaper Spectral Estimation in the Chronux Toolbox (http://chronux.org/) (Figures S1A and S1G). Coherence spectra between OB and other brain areas was calculated by using coherency function, which was based on the Multitaper coherency method from Chronux toolbox as well (Figures S1B–S1D). For all the analysis described above 3 s sliding windows with a 50% overlap were used.^83^ To test the correlation between gamma power and sucrose consumption in the optogenetic experiments, gamma power change was defined as [(PirC averaged gamma power from 10 min after illumination – PirC averaged gamma power from 10 min before illumination)/ PirC averaged gamma power from 10 min before illumination] (Figures 2G –2J).

#### Off-line analysis of gamma events

Off-line detection of the gamma activity was performed in the acute CNO mouse/rat experiments (Figures 1E and S2) and closed-loop gamma e-stim experiments (Figures 3, 4, S8, S9, and S10). First, the LFP was band-pass filtered with a eighth order zero phase lag Butterworth filter at 30–80 Hz, and RMS power was calculated in 50 ms sliding windows. Outliers of pooled power values were removed to obtain the mean and standard deviation of power values as reference. Gamma bursts were detected where the power values exceeded 3 times of standard deviation (S.D.) above the mean value for that particular frequency for at least three consecutive windows. The boundaries of each gamma events were determined where the power values fell below mean + 2 S.D. around the previously identified peaks. Detection accuracy was confirmed by subjective visual observation. For the analysis of gamma incidence in the closed-loop gamma e-stim experiment, awake periods were detected from the first 2 h of the home cage recordings.

#### Statistical analysis

Data are presented as mean ± S.D (Table S1). Statistical testing was performed using MATLAB. In the chemogenetics experiments, two-way repeated ANOVA followed by Tukey’s post hoc test was employed to compare behavioral performance in the chronic mice CNO experiment between the mCherry and the hM4Di groups. Wilcoxon rank-sum test was employed to compare gamma power changes between the two groups in the acute CNO mouse/rat experiments. In the optogenetics experiments, Pearson’s correlation coefficient was used for testing the correlation between performance of SPT and reduction of gamma oscillations in the PirC and OB, respectively. One-way ANOVA followed by Tukey’s post hoc test was employed to examine SPT among 24 h, WD + 2 h and WD + light +2 h in the optogenetic experiments, and among three phase-dependent electrical stimulus in the closed-loop gamma e-stim experiments and also for gamma incidence in the latter experiments. Wilcoxon signed-rank test was used for OFT and EPM test in the both optogenetics experiments and the closed-loop experiments. Unpaired *t*-test was employed for power changes of gamma events in the OBx experiments, the closed-loop experiments and the CNO water consumption. In the ketamine experiments, one-way ANOVA test followed by Tukey’s post hoc test was employed to examine SPT, and Wilcoxon rank-sum test was used for testing the significance of EPM behavioral test and the gamma power changes in multi-brain areas. In the LPS experiments, unpaired *t*-test was employed to test SPT performance, and Wilcoxon rank-sum test was used for testing the significance of other behavioral tests such as OFT and EPM. The significance level was set at *P* < 0.05. ^∗^, ^∗∗^ and ^∗∗∗^ indicate differences of *P* < 0.05, *P* < 0.01 and *P* < 0.001, respectively. Details are shown in Table S2).

## Data Availability

All data reported in this paper will be shared by the lead contact upon request. The custom-written code for detection of gamma events used in this study has been deposited at Zenodo and is publicly available as of the date of publication. DOI is listed in the key resources table. Any additional information required to reanalyze the data reported in this paper is available from the lead contact upon request.

## Supplemental information

Document S1. Figures S1–S11 and Tables S1 and S3–S5

Table S2. Statistical table, related to Figures 1, 2, 3, 4, and 5 Provided as a separate excel file.

Document S2. Article plus supplemental information
