# Supplementary material for: Reinstating olfactory bulb-derived limbic gamma oscillations alleviates depression-like behavioral deficits in rodents
Source: Neuron. 2023 Jul 5;111(13):2065–2075.e5. doi: 10.1016/j.neuron.2023.04.013 (PMC10321244; doi:10.1016/j.neuron.2023.04.013)
Supplement: Document S1. Figures S1–S11 and Tables S1 and S3–S5 [file mmc1.pdf]

Neuron, Volume *111*

Supplemental information

Reinstating olfactory bulb-derived limbic gamma oscillations alleviates depression-like behavioral deficits in rodents

Qun Li, Yuichi Takeuchi, Jiale Wang, Levente Gellért, Livia Barcsai, Lizeth K. Pedraza, Anett J. Nagy, Gábor Kozák, Shinya Nakai, Shigeki Kato, Kazuto Kobayashi, Masahiro Ohsawa, Gyöngyi Horváth, Gabriella Kékesi, Magor L. Lőrincz, Orrin Devinsky, György Buzsáki, and Antal Berényi

Supplementary Figures

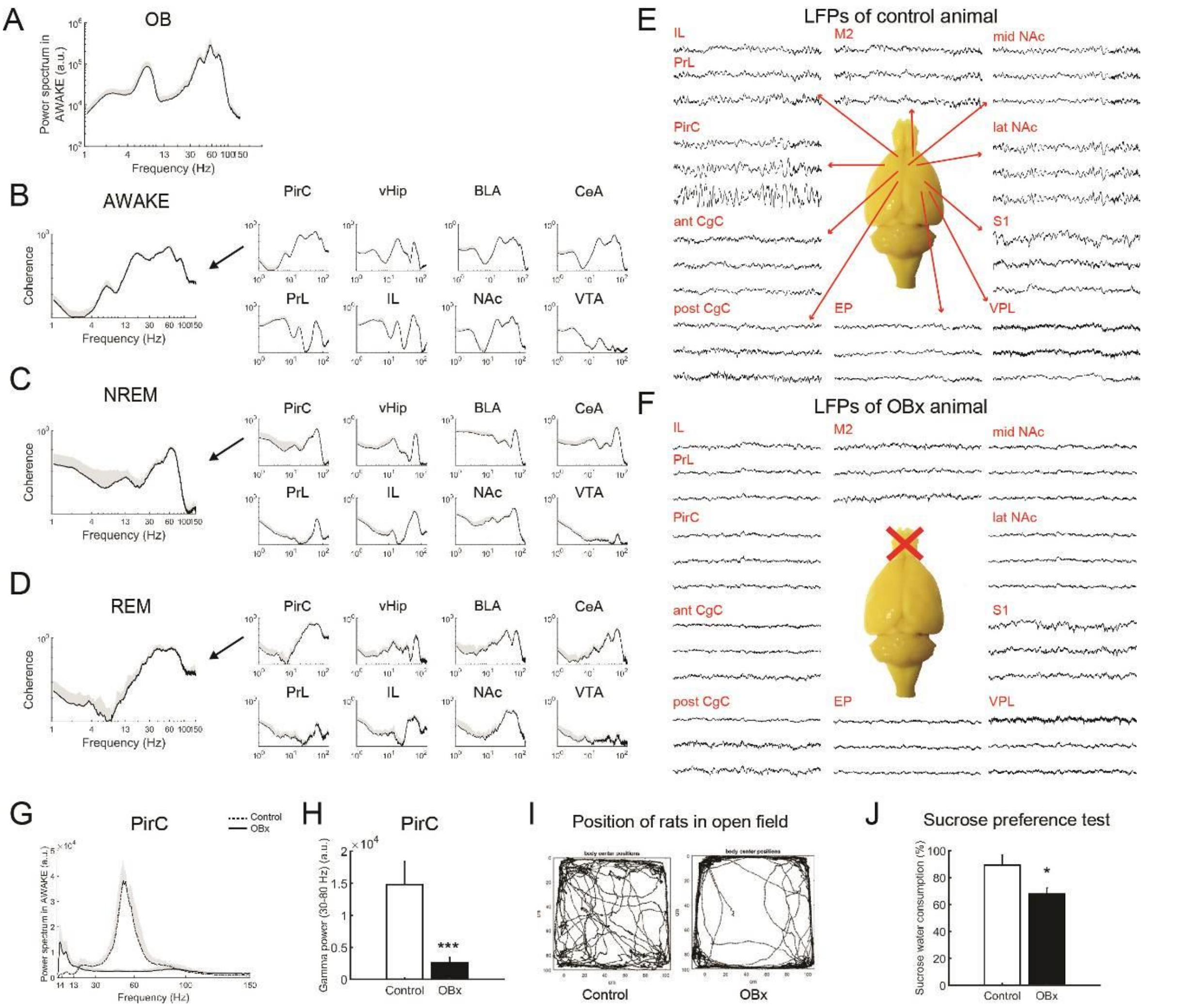

Figure S1. Olfactory bulbectomy reduces global gamma oscillations and induces depression-like behaviors in rats. Related to Figure 1. (**A**) Power spectrum of OB local field potentials (LFPs) in intact animals during the AWAKE state. OB, olfactory bulb. (**B–D**) Highly coherent gamma oscillations between the OB and multiple brain regions during the AWAKE (B), NREM (C) and REM (D) states, respectively. PirC, piriform cortex; vHip, ventral hippocampus; CeA/BLA, central amygdala/basolateral amygdala; PrL/IL, prelimbic cortex/ infralimbic cortex; VTA, ventral tegmental area. (**E**, **F**) Representative LFPs of a control rat (E) and an OBx rat (F) in multiple brain regions. M2, secondary motor cortex; mid NAc, medial nucleus accumbens; lat NAc, lateral nucleus accumbens; ant CgC, anterior cingulate cortex; post CgC, posterior cingulate cortex; S1, primary somatosensory cortex; EP, entopeduncular nucleus; VPL, ventral posterolateral thalamic nucleus. (**G**) Power spectrum in the PirC of control animals (dashed line) and OBx animals (bold line) during the AWAKE state. Grey shadow indicates S.D. (**H**) Statistical results in the gamma band corresponding to (G). (**I**) Representative traces of control and OBx animals' position in the open field in 10 mins one month after OBx. (**J**) Percentage of sucrose water consumption in both of the groups one month after the surgery. (n = 3 rats/ group). Values are represented as means + S.D. *** indicates $P < 0.0001$.

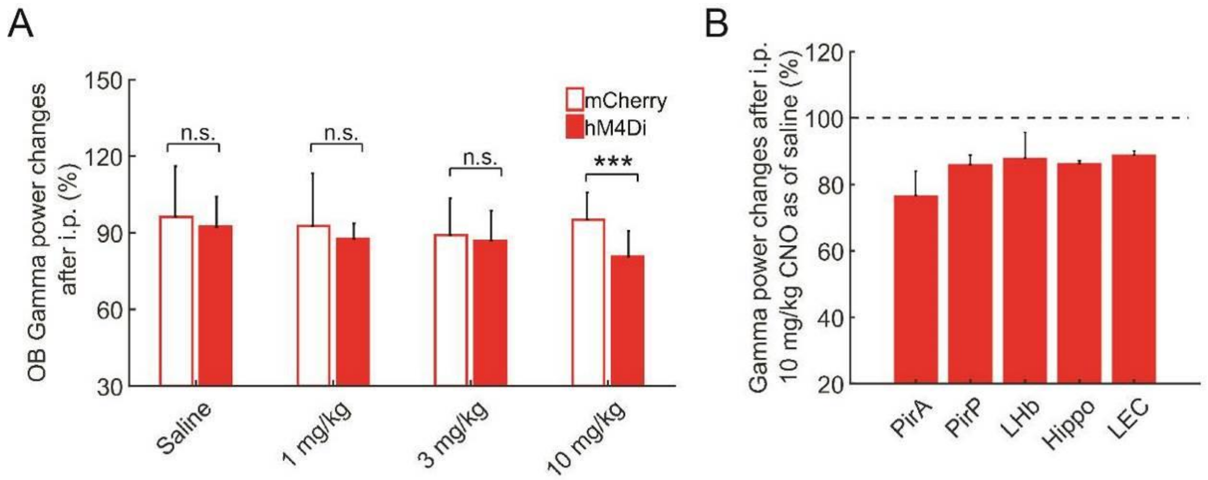

Figure S2. Chemogenetic inhibition of OB neurons decreases OB gamma oscillations in a dose dependent manner in rats. Related to Figure 1. (**A**) Changes of OB gamma oscillations (30–80 Hz) after systemic administration of either saline or CNO in both of mCherry and hM4Di-mCherry groups. The protocol is the same as the four days long acute CNO experiments on mice (Figure 1D). (**B**) Representative brain wide power changes of gamma oscillations after systemic administration of 10 mg/kg CNO in hM4Di rat. Values are represented as means + S.D. n.s., not significant; ***$P < 0.001$.

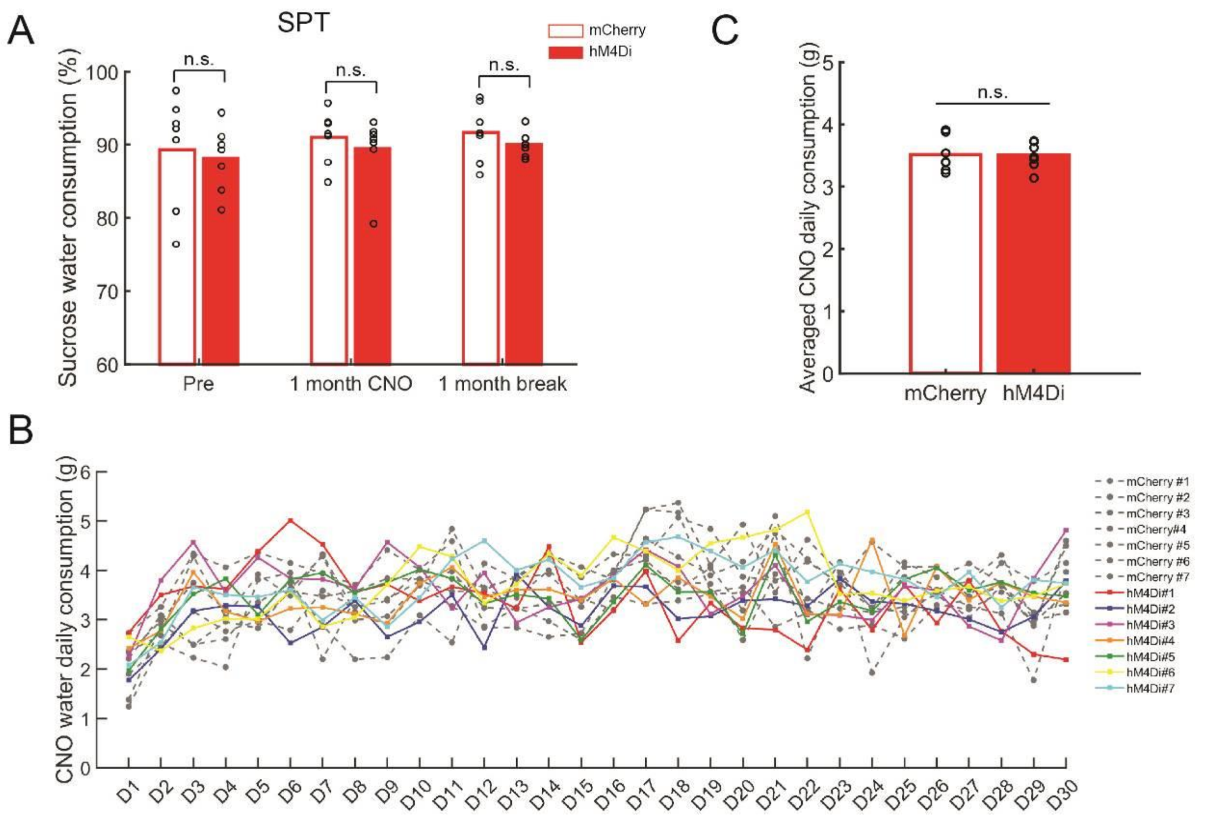

Figure S3. Chemogenetic inhibition of OB neurons doesn't change the consumption of either CNO water or sucrose water. Related to Figure 1. (**A**) No significant differences were found between the CNO treated hM4Di and control groups in the sucrose preference test (SPT) (n = 7 mice / group). (**B**) Individual daily CNO solution consumption of the animals. Each line represents one mouse. (**C**) No significant differences were found in the averaged CNO daily consumption (n = 7 mice / group). Circles and bars denote per animal averages and means across animals, respectively. n.s., not significant.

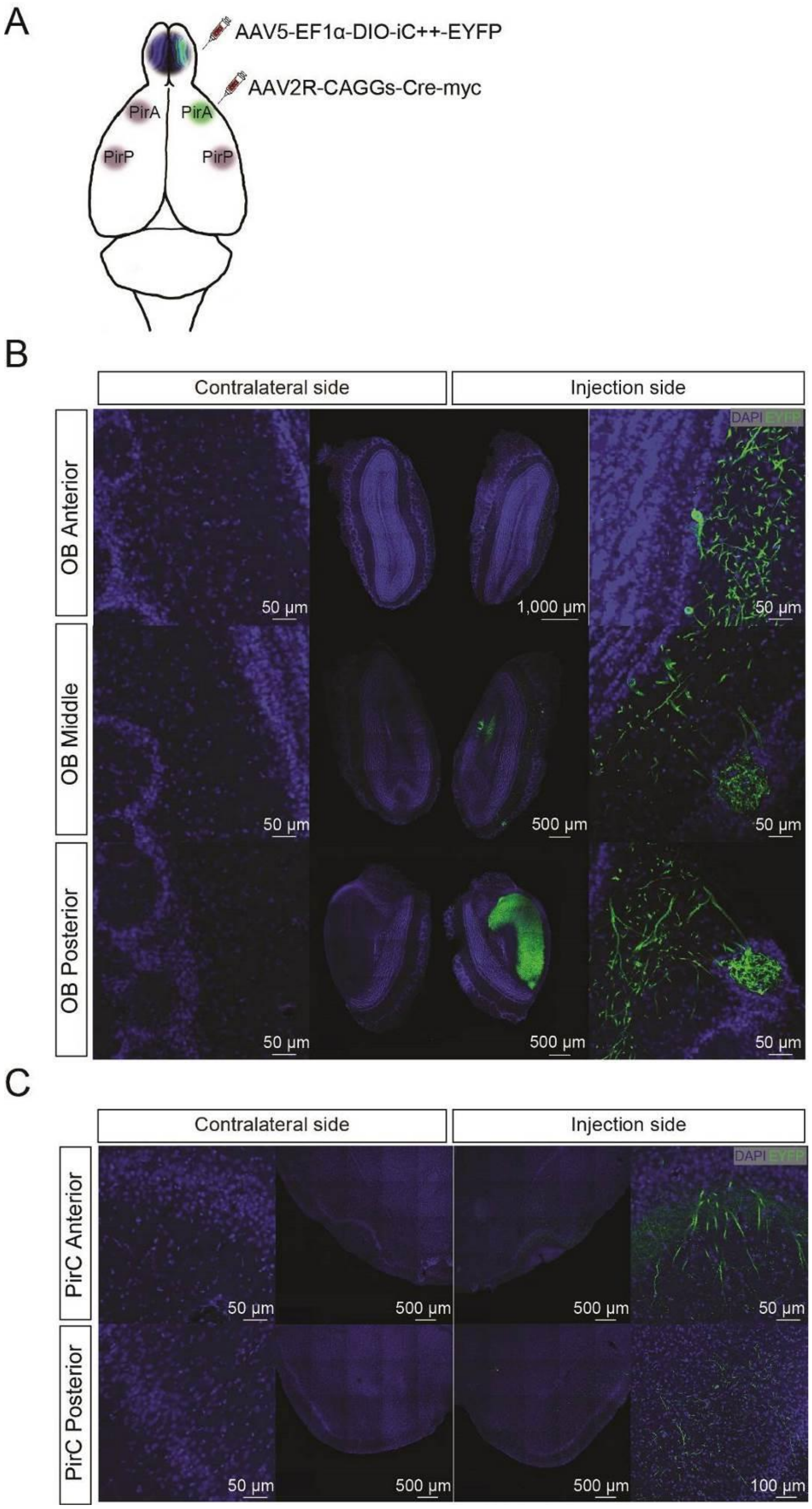

Figure S4. Visualization of OB neurons projecting to the anterior part of the ipsilateral PirC. Related to Figure 2. (**A**) The schema of viral vector injections. (**B**, **C**) EYFP expression is present ipsilateral to the injection sites in the OB (**B**) and the PirC (**C**), respectively, but not in the contralateral side.

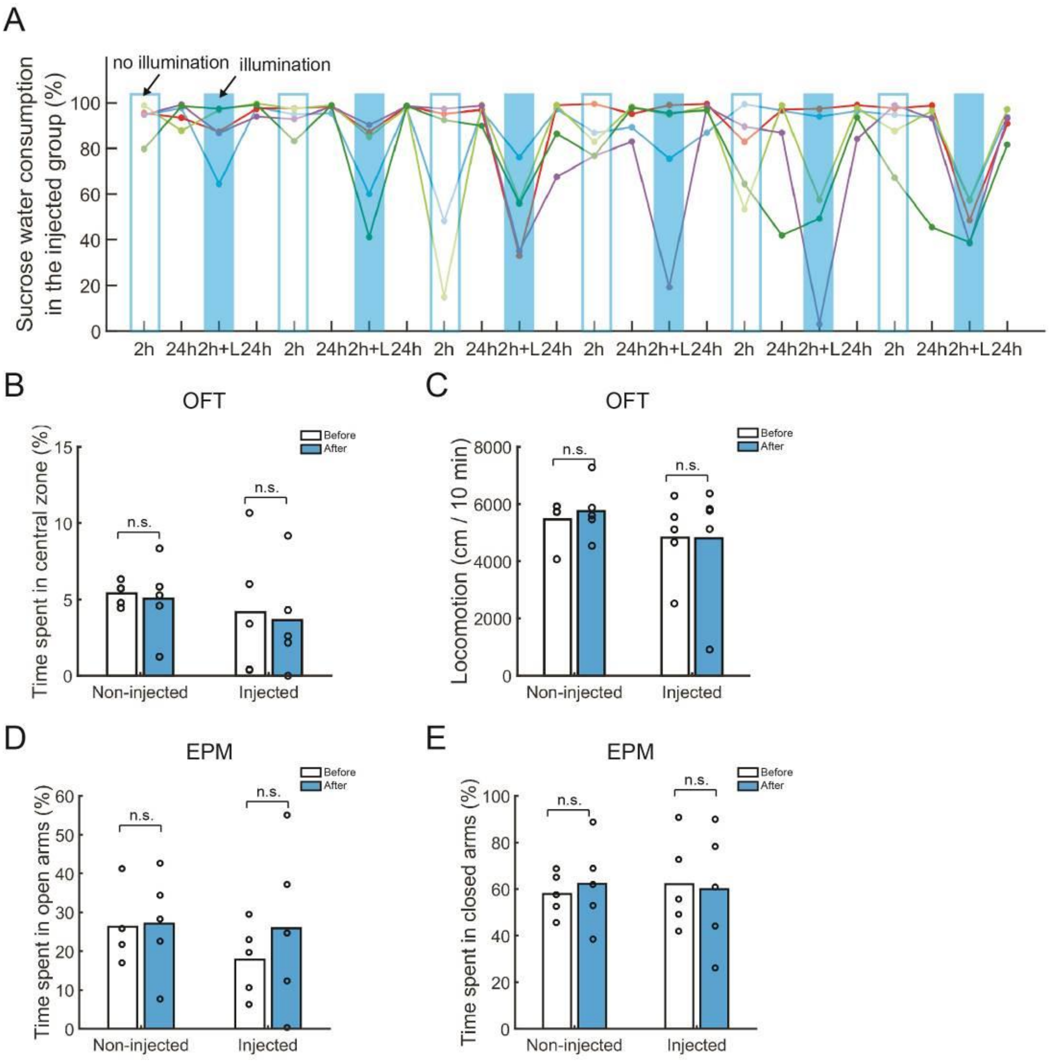

Figure S5. Behavioral performances during and after the selective, reversible suspension of synaptic transmission of the OB to PirC pathway by optogenetic CALI (InSynC). Related to Figure 2. (**A**) Time courses of sucrose preference of individual rats in the injected group during the whole protocol as showed in Figure 2E. Colored lines and markers indicate individual rats (n = 5). Open blue and solid blue bars mark test sessions with and without illuminations, respectively. 2 h, two hour sucrose preference (SPT) test after 22 hours water deprivation; 24 h, 24 hours SPT without water deprivation; L, light/optostimulation. (**B**, **C**) No significant differences were found in the either groups either in the time spent in the central zone (**B**) or locomotion (**C**) in the open field test (OFT), comparing before illumination (Before) and after illumination (After) measurements. (**D, E**) No significant differences were found in the either groups either in the time spent of open arms (**D**) or closed arms (**E**) in the elevated plus maze (EPM) test comparing before illumination and after illumination periods (n = five rats / group). Circles and bars denote per animal and across animal averages, respectively. n.s., not significant.

A

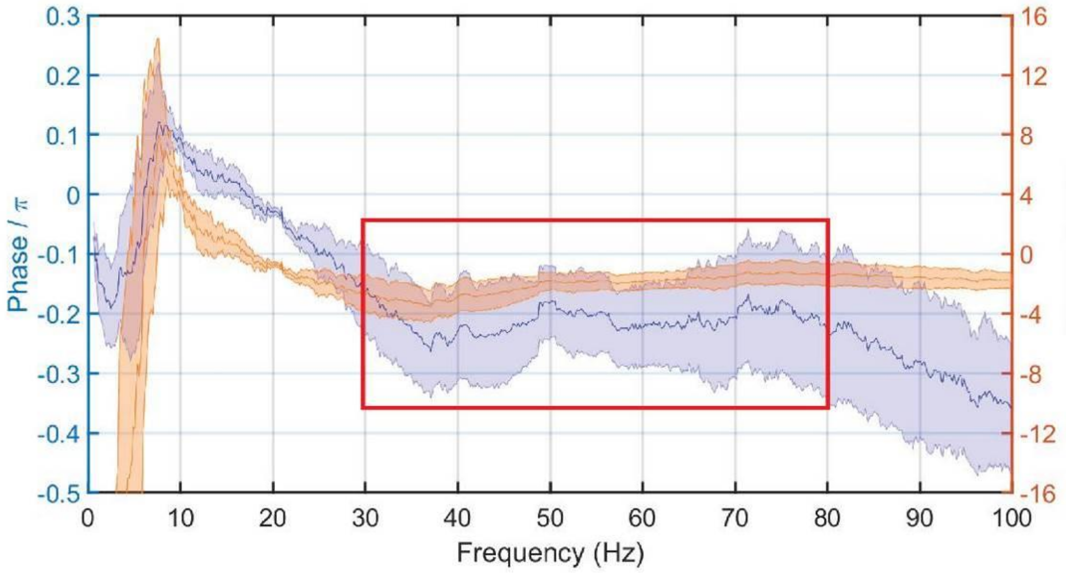

Figure S6. Phase lag coherency between the OB and the PirC in naïve rats during AWAKE state. Related to Figure 3. (**A**) Phase lag coherency between the OB and the PirC from 0–100 Hz with a window width of 5 s during 10–30 min AWAKE state. The gamma band (30–80 Hz) of PirC signal is lagging OB activity with a -0.21π ± 0.08 π. (n = five rats). Blue color is Phase lag coherence (chronux toolbox) in radians. Orange color is phase lag calculated by extracted gamma events (see the Methods section, Off-line analysis of gamma events). Shading denotes S.D. from the five rats.

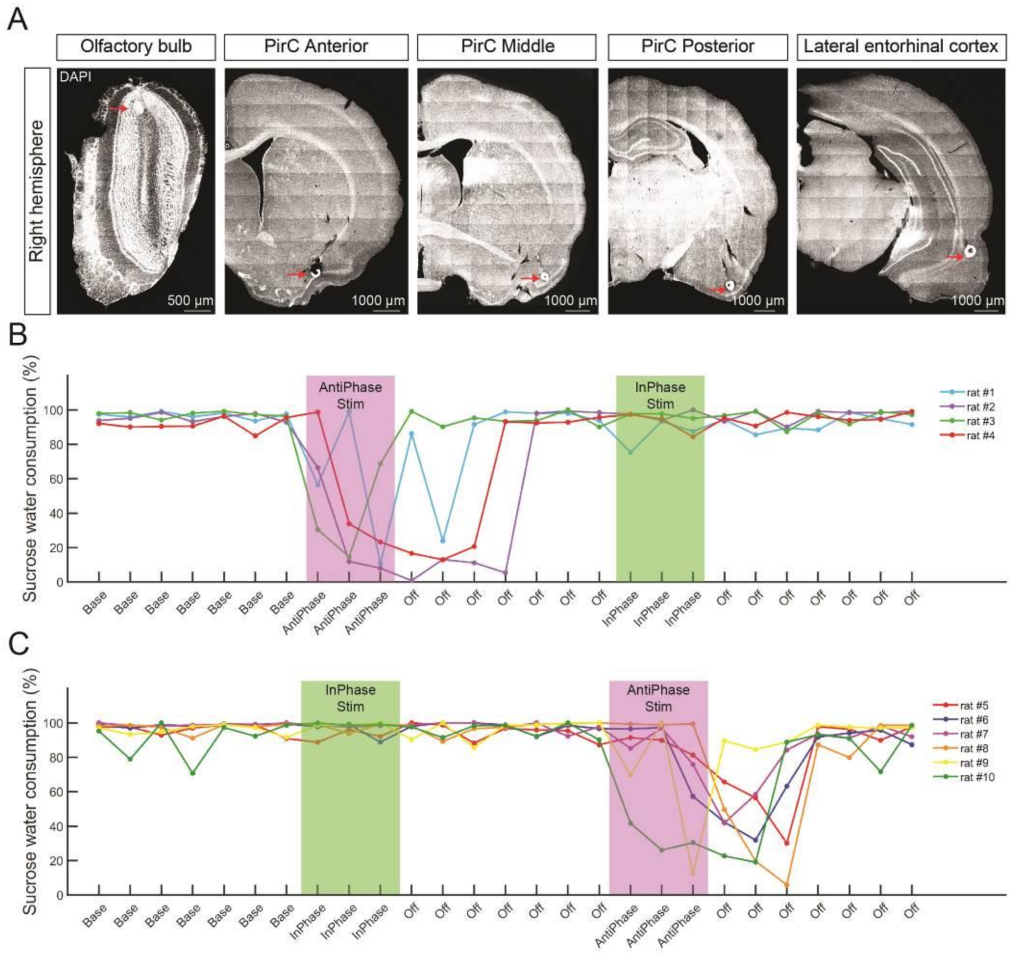

Figure S7. Time courses of sucrose water consumption of individual rats with real-time closed-loop feed of OB gamma oscillations to the PirC. Related to Figure 3. (**A**) Post-mortem identification of recording sites' locations. Each arrow indicates a recording site. (**B**) Time course of sucrose water consumption of the four rats that underwent the AntiPhase - InPhase protocol as shown in Figure 3C. Colored lines denote individual rats. (**C**) Time course of sucrose water consumption of the six rats that underwent the flipped sequence of InPhase - AntiPhase stimulation. The order of stimulation paradigms did not affect their performance.

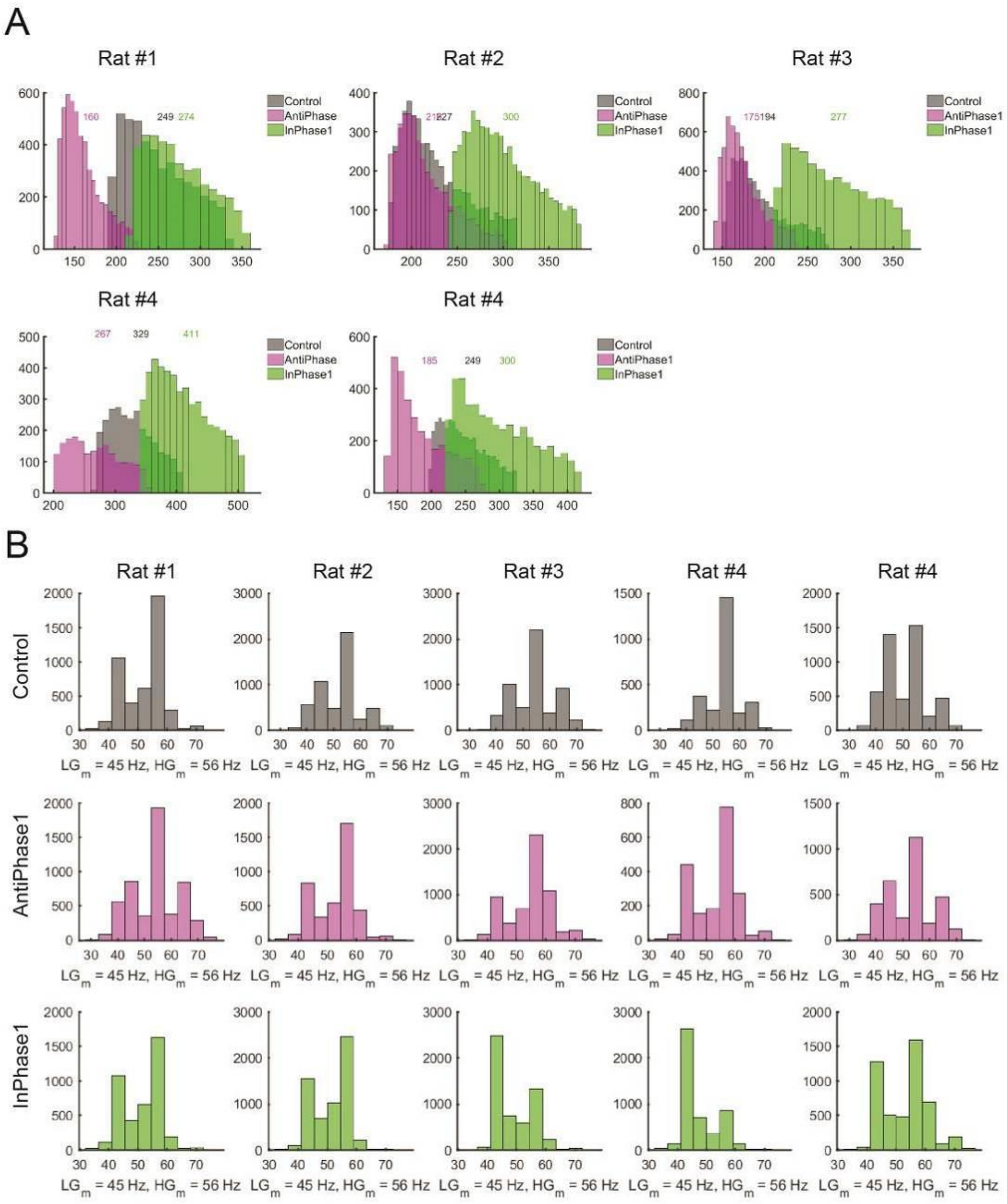

Figure S8. Features of gamma events in the PirC after real-time closed-loop feed of the OB gamma oscillations to PirC. Related to Figure 3. (**A**) The power distribution of gamma events in each individual trial during one hour LFP recording during Baseline (grey), during the day after AntiPhase stimulation (magenta) and the day after InPhase stimulation (green). The numbers in each figure represent medians of the distributions. The conventions are the same as Figure 3H. (**B**) The frequency distributions of gamma events of individual trials shown in (A). LG_m represents the median of frequency from 30 to 50 Hz, and HG_m represents the medians of frequency from 50 to 80 Hz. Five trials from four rats are as shown in Fig S7 B.

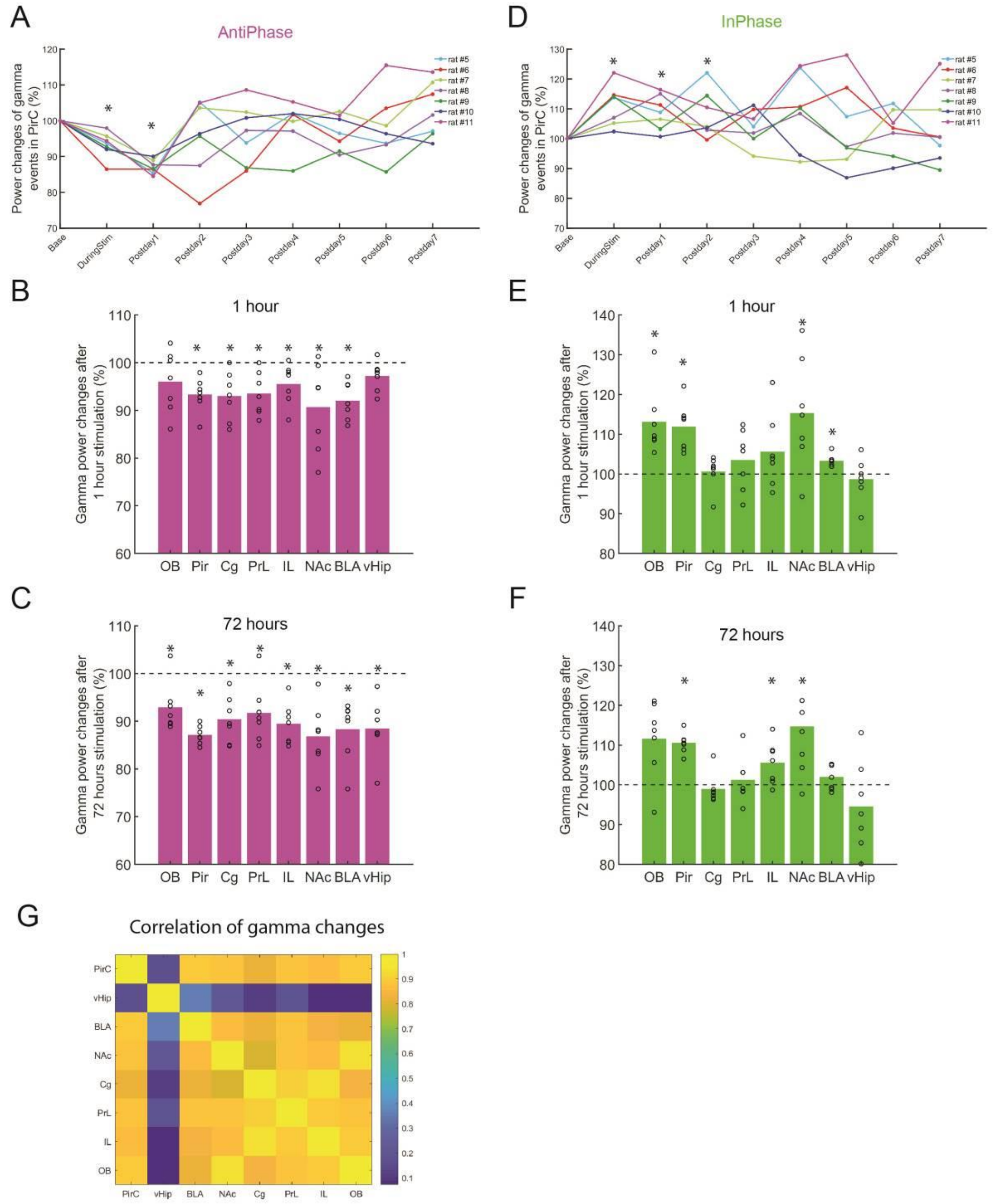

Figure S9. Gamma power changes in the multi brain areas during and after stimulation. Related to Figure 3. (**A**) and (**D**) Time courses of gamma power changes in the PirC of individual rats following AntiPhase (A) and InPhase (D) stimulation. (n = 7 rats per group). (**B**) and (**E**) Gamma power changes in multi brain areas after 1 h AntiPhase (B) and InPhase (E) stimulation. (**C**) and (**F**) Gamma power changes in multi brain areas after 72 h AntiPhase (C) and InPhase (F) stimulation. (**G**) Correlation map of gamma power changes in multi brain areas during whole recording procedure. (n = 134 trials from four rats). Circles and bars indicate individual trials and means, respectively. n.s. indicates not significant difference. * indicates difference of $P < 0.05$.

A

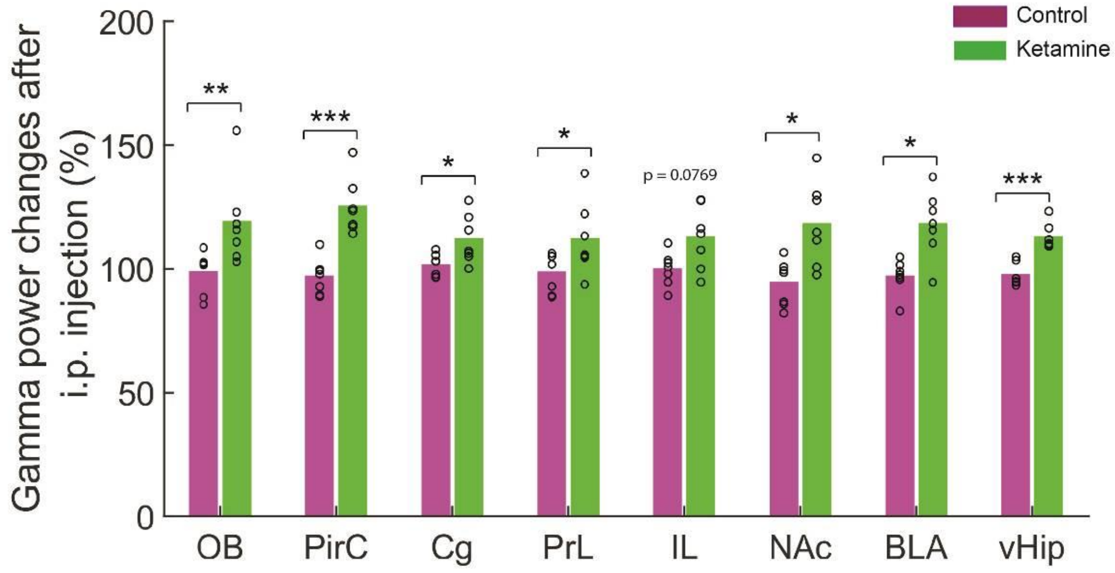

Figure S10. Gamma power changes in the multi brain areas after ketamine administration. Related to Figure 4. (**A**) Gamma power increased in the most limbic brain areas after the ketamine administration. (n = seven rats / group). Circles and bars represent individual trials and means, respectively. *, **, and *** indicate difference of $P < 0.05$, $P < 0.01$, and $P < 0.001$, respectively.

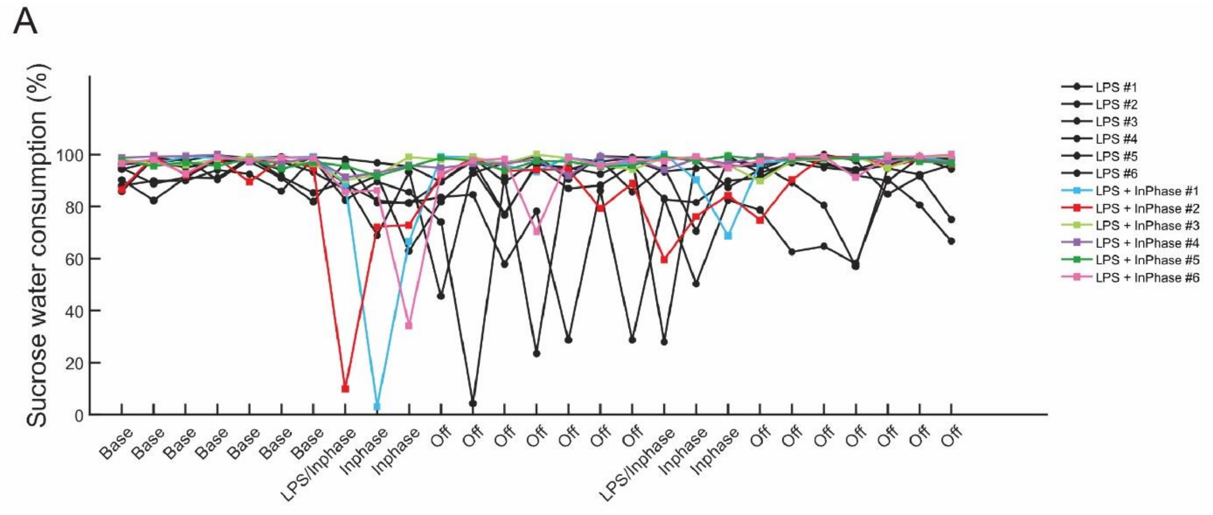

Figure S11. Time courses of sucrose water consumption during the InPhase stimulation. Related to Figure 5. (**A**) Time courses of sucrose water consumption of individual rats following two sessions of the systemic LPS administrations. Related to the Figure 5B group data. (n = six rats per group)

Supplementary Tables

Table S1. Results description table. *Related to STAR Methods - #Statistical analysis.*

Paragraph	Panel	Claim/Conclusion	Supporting data & Statistics
**5**	**Figures S1G and S1H**	… gamma oscillations were markedly attenuated in OBx rats compared to controls.	Gamma power: 14751.6 ± 3630.9 vs 2662.5 ± 778.4, *P* < 0.0001, unpaired *t*-test, 10 min with 5 s window length for each session, in total 12 sessions from three intact rats and three OBx rats
**5**	**Figure S1I**	… including signs of anxiety …	7.1 ± 3.7 % vs 1.4 ± 0.7 %, *P* < 0.05,<br>Wilcoxon rank-sum test
**5**	**Figure S1J**	… and anhedonia …	89.3 ± 7.6 % vs 67.8 ± 4.7 %, *P* < 0.05,<br>Wilcoxon rank-sum test
**6**	**Figures 1D and 1E**	After systemic CNO administration, OB gamma power (30-80 Hz) was suppressed in a dose-dependent manner in both mice and rats.	Saline: 91.5 ± 9..0 % vs 93.7 ± 8.3 %, *P* = 0.1552;<br>1 mg/kg CNO: 92.5 ± 8.8 % vs 89.8 ± 6 %, *P* = 0.0852;<br>3 mg/kg CNO: 90.1 ± 13.4 % vs 80.6 ± 10.2 %, *P* = 0.0757;<br>10 mg/kg CNO: 92.7 ± 5 % vs 51.1 ± 8.6 %, *P* < 0.001;<br>Wilcoxon rank sum test, 5 min for each trial, in total 240 trials from three mCherry and three hM4Di mice, respectively.
**6**	**Figure S2A**		Saline: 96.2 ± 19.8 % vs 92.3 ± 11.7 %, *P* = 0.3859;<br>1 mg/kg CNO: 92.6 ± 20.5 % vs 87.7 ± 5.9 %, *P* = 0.2727;<br>3 mg/kg CNO: 89.1 ± 14.4 % vs 86.9 ± 11.5 %, *P* = 0.4828;<br>10 mg/kg CNO: 95.2 ± 10.7 % vs 80.6 ± 10.1 %, *P* < 0.001;<br>Wilcoxon rank sum test, 5 min for each trial, in total 160 and 320 trials from two mCherry and four hM4Di rats, respectively
**6**	**Figure 1H**	The hM4Di group showed anxiety-like behavior with less time spent in the open field center …	Two-way repeated ANOVA,<br>main effect of group, *F* (1, 41) = 5.7920, *P* < 0.05;<br>main effect of time, *F* (2, 41) = 9.9245, *P* < 0.001;<br>interaction, *F* (2, 41) = 0.7410, *P* = 0.4838;<br>Tukey's post hoc test,<br>Pre: 11.7 ± 5.2 % vs 8.3 ± 4.8 %, *P* = 0.2223;<br>1 month CNO: 7.5 ± 1.9 % vs 3.5 ± 2.2 %, *P* < 0.01;<br>1 month break: 4.5 ± 3.7 % vs 3.6 ± 3.2 %, *P* = 0.6664
**6**	**Figure 1G**	… but no reduction in locomotion.	Two-way repeated ANOVA,<br>main effect of group, *F* (1, 41) = 0.8197, *P* = 0.3713;<br>main effect of time, *F* (2, 41) = 0.8049, *P* = 0.4550;<br>interaction, *F* (2, 41) = 0.2243, *P* = 0.8002;<br>Pre: 4036.6 ± 576.8 cm vs 4219.4 ± 1780.1 cm;<br>one month CNO: 3535.3 ± 852.9 cm vs 3690.4 ± 1518 cm;<br>one month break: 3229.4 ± 882.1 cm vs 3953.4 ± 1523.2 cm
**6**	**Figure S3A**	… sucrose preference test (SPT) nor daily liquid consumption	Two-way repeated ANOVA,<br>main effect of group, *F* (1, 41) = 1.0279, *P* = 0.3174;<br>main effect of time, *F* (2, 41) = 0.7538, *P* = 0.4779;<br>interaction, *F* (2, 41) = 0.0085, *P* = 0.9916;<br>Pre: 89.3 ± 7.7 % vs 88.1 ± 4.5 %;<br>one month CNO: 91.0 ± 3.6 % vs 89.4 ± 4.7 %;<br>one month break: 91.7 ± 4.0 % vs 90.0 ± 1.7 %

**6**	**Figures S3B and S3C**		3.52 ± 0.72 g vs 3.50 ± 0.61 g, *P* = 0.9187, unpaired t-test
**8**	**Figure 2F**	Following bilateral PirC PS, the animals showed reduced performance in the SPT …	One-way ANOVA, Non-Injected group: *F* (2, 119) = 1.07, *P* = 0.3451; 24 h: 91.5 ± 9.8 %, WD + 2 h SPT: 89.2 ± 11.1 %, WD + Light + 2 h SPT: 88.2 ± 12.7 %; Injected group: *F* (2, 119) = 23.1645, *P* < 0.0001; 24 h vs WD + 2 h SPT: 93.3 ± 10.9 % vs 84.6 ± 18.9 %, *P* = 0.0791; WD + 2 h SPT vs WD + Light + 2 h SPT: 84.6 ± 18.9 % vs 66.0 ± 26.5 %, *P* < 0.0001, Tukey's post hoc test
**8**	**Figure 2G**	Sucrose consumption was positively correlated with gamma power in the PirC	Pearson's correlation test, *P* < 0.001
**8**	**Figure 2H**	… but not OB	Pearson's correlation test, *P* = 0.113
**8**	**Figure 2I**	Photostimulation failed to affect these functions in naïve animals …	Pearson's correlation test, *P* = 0.2766
**8**	**Figure 2J**		Pearson's correlation test, *P* = 0.6924
**8**	**Figures S5Band S5C**	No significant changes were found in OFT … following PirC PS	Wilcoxon signed rank test, Non-injected group: Time spent in the center of OFT: 5.4 ± 0.8 % vs 5.1 ± 2.6 %, *P* = 0.8125, Figure S5B; Locomotion in OFT: 5470.7 ± 786.4 cm/10 min vs 5753.1 ± 996.5 cm/10 min, *P* = 0.6250, Figure S5C; Injected group: Time spent in the center of OFT: 4.2 ± 4.3 % vs 3.6 ± 3.4 %, *P* = 1, Figure S5B; Locomotion in OFT: 4831.6 ± 1422.9 cm/10 min vs 4801.2 ± 2219.7 cm/10 min, *P* = 0.8125, Figure S5C;
**8**	**Figures S5D and S5E**	No significant changes were found in … Elevated Plus Maze (EPM) test following PirC PS	Wilcoxon signed rank test, Non-injected group: Time spent in open arms of EPM: 26.3 ± 9.1 % vs 27.1 ± 13.2 %, *P* = 1, Figure S5D; Time spent in closed arms of EPM: 57.9 ± 9.3 % vs 62.2 ± 18.7 %, *P* = 0.6250, Figure S5E; Injected group: Time spent in open arms of EPM: 17.8 ± 9.4 % vs 25.9 ± 21.3 %, *P* = 0.3125, Figure S5D; Time spent in closed arms of EPM: 62.1 ± 19.7 % vs 59.9 ± 25.7 %, *P* = 0.6250, Figure S5E
**9**	**Figures 3D and S7**	AntiPhase gamma E-Stim (i.e. interfering with PirC rhythmic neuronal activity) decreased sucrose preference in all animals and the effect outlasted the stimulation	One-way ANOVA, *F* (4, 90) = 28.3743, *P* < 0.0001;<br>Base vs AntiPhase: 95.4 ± 3.1 % vs 49.4 ± 35.8 %, *P* < 0.0001;<br>Base vs AntiPhaseOff: 95.4 ± 3.1 % vs 42.2 ± 37.5 %, *P* < 0.0001;<br>InPhase: 92.7 ± 6.6 %, *P* = 0.9931;<br>InPhaseOff: 93.9 ± 4.4 %, *P* = 0.9994;<br>Tukey's post hoc test
**9**	**Figure 3E**	Neither InPhase nor AntiPhase E-Stim affected the rats' spontaneous locomotion in their homecage	One-way ANOVA, *F* (2,62) = 0.3323, *P* = 0.7186;<br>Control: 1136.2 ± 376.5 cm/h,<br>AntiPhase: 1057.5 ± 267.2 cm/h,<br>InPhase: 1138.5 ± 263.5 cm/h
**9**	**Figure 3F**	AntiPhase E-Stim induced depression-like symptoms (i.e., decreased time spent in central zone of OFT …	Wilcoxon rank-sum test,<br>Control vs AntiPhase: 6.0 ± 2.8 % vs 3.3 ± 2.7 %, *P* < 0.05<br>AntiPhase vs InPhase: 3.3 ± 2.7 % vs 3.3 ± 4.3 %, *P* < 0.05,
**9**	**Figure 3G**	… and in open arms of EPM test.	Wilcoxon rank-sum test,<br>Control vs AntiPhase: 28.0 ± 14.2 % vs 16.6 ± 14.3 %, *P* < 0.05<br>AntiPhase vs InPhase: 16.6 ± 14.3 % vs 28.5 ± 21.3 %, *P* < 0.05,

**9**	**Figures 3H, 3I and S8A**	These changes persisted for one day after stimulations.	AntiPhase: 81.6 ± 15.9%, P < 0.001; InPhase: 128.7 ± 14.6 %, P <0.001 normalized to the Base power of each trial; t-test
**9**	**Figure 3J**	… and gamma events incidence during wakefulness was unaltered.	One-way ANOVA, F (2,147) = 1.3521, P = 0.2619; Base: 93 ± 17 / min; AntiPhase: 94 ± 25 / min; InPhase: 88 ± 21 / min
**9**	**Figure S9A**	AntiPhase time course PirC	Wilcoxon signed rank test, Base vs DuringStim: 100 ± 0 % vs 93.3 ± 3.6 %, P < 0.05; Base vs PostDay1: 87.1 ± 1.9 %, P < 0.05; Base vs PostDay2: 95.8 ± 10.5 %, P = 0.8215; Base vs PostDay3: 96.5 ± 8.3 %, P = 0.3281; Base vs PostDay4: 99.1 ± 6.3 %, P = 0.8125; Base vs PostDay5: 96.7 ± 4.9 %, P = 0.2188; Base vs PostDay6: 98.1 ± 9.4 %, P = 0.4688; Base vs PostDay7: 102.9 ± 7.7 %, P =0.5781;
**9**	**Figure S9B**	AntiPhase 1 hours in multi-brain areas	Wilcoxon signed rank test, OB: 96.0 ± 6.5 %, P = 0.2969; PirC: 93.3 ± 3.6 %, P < 0.05; Cg: 93.0 ± 5.1 %, P < 0.05; PrL: 93.5 ± 4.5 %, P < 0.05; IL: 95.5 ± 4.3 %, P < 0.05; NAc: 90.7 ± 9.2 %, P < 0.05; BLA: 92.0 ± 4.0 %, P < 0.05; vHip: 97.2 ± 3.1 %, P = 0.2188;
**9**	**Figure S9C**	AntiPhase 72 hours in multi-brain areas	Wilcoxon signed rank test, OB: 92.9 ± 5.1 %, P < 0.05; PirC: 87.1 ± 1.9 %, P < 0.05; Cg: 90.4 ± 4.8 %, P < 0.05; PrL: 91.7 ± 6.2 %, P < 0.05; IL: 89.4 ± 4.4 %, P < 0.05; NAc: 86.8 ± 6.9 %, P < 0.05; BLA: 88.3 ± 6.3 %, P < 0.05; vHip: 88.4 ± 6.2 %, P < 0.05;
**9**	**Figure S9D**	InPhase time course PirC	Wilcoxon signed rank test, Base vs DuringStim: 100 ± 0 % vs 111.3 ± 6.8 %, P < 0.05; Base vs PostDay1: 108.8 ± 5.8 %, P < 0.05; Base vs PostDay2: 108.2 ±7.9 %, P < 0.05; Base vs PostDay3: 103.9 ± 5.9 %, P = 0.3125; Base vs PostDay4: 109.2 ± 12.6 %, P = 0.1563; Base vs PostDay5: 103.8 ± 14.5 %, P = 0.5781; Base vs PostDay6: 102.3 ± 7.9 %, P = 0.4688; Base vs PostDay7: 102.4 ± 11.9 %, P =0.8125;
**9**	**Figure S9E**	InPhase 1 hours in multi-brain areas	Wilcoxon signed rank test, OB: 113.1 ± 8.5 %, P < 0.05; PirC: 111.8 ± 6.8 %, P < 0.05; Cg: 100.6 ± 4.1 %, P = 0.4063; PrL: 103.5 ± 7.6 %, P = 0.2188; IL: 105.6 ± 9.4 %, P = 0.2188; NAc: 115.3 ± 14.0 %, P < 0.05; BLA: 103.2 ± 1.6 %, P < 0.05; vHip: 98.7 ± 5.3 %, P = 1;
**9**	**Figure S9F**	InPhase 72 hours in multi-brain areas	Wilcoxon signed rank test, OB: 111.6 ± 9.7 %, P = 0.0781; PirC: 110.5 ± 2.6 %, P < 0.05; Cg: 98.9 ± 3.8 %, P = 0.2813; PrL: 101.2 ± 5.8 %, P = 0.8215; IL: 105.5 ± 5.4 %, P < 0.05; NAc: 114.7 ± 13.8 %, P < 0.05; BLA: 101.9 ± 3.2 %, P = 0.2969; vHip: 94.6 ± 11.3 %, P = 0.2188;
**10**	**Figure 4B**	AntiPhase E-Stim induced anhedonia in the SPT lasting several days following stimulation in control animals	Wilcoxon rank sum test, Base, Con: 95.9 ± 4.6 % vs Ketamine: 96.1 ± 2.8 %, P = 1 ; AntiPhase, Con: 63.4 ± 34.2 % vs Ketamine: 72.3 ± 28.2 %, P = 0.5883; AntiPhaseOff, Con: 47.7 ± 32.2 % vs Ketamine: 91.0 ± 7.2 %, P < 0.001
**10**	**Figure 4C**	AntiPhase E-Stim also induced anxiety-like behaviors in the EPM test in control, but not in ketamine treated animals. (OFT)	Wilcoxon rank sum test, Base, Con: 29.4 ± 12.4 % vs Ketamine: 34.6 ± 8.5 %, P = 0.2471 ; AntiPhase, Con: 15.4 ± 11.4 % vs Ketamine: 36.0 ± 17.4 %, P < 0.05

**10**	**Figure 4D**	AntiPhase E-Stim also induced anxiety-like behaviors in the EPM test in control, but not in ketamine treated animals (EPM)	Wilcoxon rank sum test, Base, Con: 49.1 ± 15.0 % vs Ketamine: 43.9 ± 8.9 %, $P = 0.5146$; AntiPhase, Con: 64.2 ± 14.3 % vs Ketamine: 42.3 ± 15.3 %, $P < 0.05$
**10**	**Figure 4E**	… but did not alter the time spent in the center zone	Wilcoxon rank sum test, Base, Con: 21.5 ± 7.4 % vs Ketamine: 21.5 ± 5.9 %, $P = 0.9790$; AntiPhase, Con: 20.4 ±6.4 % vs Ketamine: 21.7 ± 6.0 %, $P = 0.7104$
**10**	**Figure 4F**	… and increased the total travel distance	Wilcoxon rank sum test, Base, Con: 3240.3 ± 711.7 cm vs Ketamine: 3791.7.3 ± 687.4 cm, $P = 0.3013$; AntiPhase,Con: 2736.2 ± 383.8 cm vs Ketamine: 3437.5 ± 377.4 cm, $P < 0.01$
**10**	**Figure 4G**	Ketamine increased gamma power in PirC …	Wilcoxon rank sum test, Con: 96.8 ± 7.3 % vs Ketamine: 125.2 ± 11.3 %, $P < 0.001$
**10**	**Figure S10**	… and other limbic brain areas	Wilcoxon rank sum test, OB: Con 98.7 ± 8.4 % vs Ketamine 118.8 ± 17.8 %, $P < 0.01$; PirC: Con 96.8 ± 7.3 % vs Ketamine 125.2 ± 11.3 %, $P < 0.001$; Cg: Con 101.5 ± 4.5 % vs Ketamine 111.8 ± 9.8 %, $P < 0.05$; PrL: Con 98.4 ± 7.9 % vs Ketamine 111.9 ± 14.6 %, $P < 0.05$; IL: Con 99.8 ± 6.7 % vs Ketamine 112.6 ± 12.8 %, $P = 0.0769$; NAc: Con 94.3 ± 9.3 % vs Ketamine 118.1 ± 16.9 %, $P < 0.05$; BLA: Con 96.9 ± 6.9 % vs Ketamine 118.1 ± 13.5 %, $P < 0.05$; vHip: Con 97.4 ± 4.7 % vs Ketamine 112.8 ± 5.3 %, $P < 0.001$
**11**	**Figures 5B and S11**	LPS decreased sucrose preference, but the group receiving InPhase gamma E-Stim recovered SPT perf.	unpaired t-test, LPS group: 85.6 ± 14.7 %; LPS + InPhase group: 83.7 ± 23.6 %; $P = 0.6888$; LPS group: 86.2 ± 18.6 %; LPS + InPhase group: 96.3 ± 4.9 %; $P < 0.0001$
**11**	**Figure 5C**	InPhase E-Stim also increased the 'center time' during the OFT	Wilcoxon rank-sum test, LPS group: 3.9 ± 2.3 %; LPS + InPhase group: 6.6 ± 2.9 %; $P < 0.05$,
**11**	**Figure 5D**	… number of center entries ...	Wilcoxon rank-sum test, LPS group: 6.5 ± 3.7; LPS + InPhase group: 10.4 ± 3.6; $P < 0.01$
**11**	**Figure 5E**	… and distance travelled per time unit …	Wilcoxon rank-sum test, LPS group: 6.9 ± 1.3 cm/s; LPS + InPhase group: 9.1 ± 2.0 cm/s; $P < 0.01$
**11**	**Figure 5F**	InPhase gamma E-Stim alleviated anxiety-like behaviors in the EPM test …	Wilcoxon rank-sum test, LPS group: 15.4 ± 13.0 %; LPS + InPhase group: 29.4 ± 17.8 %; $P < 0.05$
**11**	**Figure 5I**	… and increased distance travelled per time unit …	Wilcoxon rank sum test, LPS group: 4.3 ± 0.6 %; LPS + InPhase group: 5.0 ± 1.1 %; $P < 0.05$
**11**	**Figure 5G**	… but did not alter the time in closed arms…	Wilcoxon rank-sum test, LPS group: 64.3 ± 19.1 %; LPS + InPhase group: 53.8 ± 18.8 %; $P = 0.0921$,
**11**	**Figure 5H**	… or in the center	Wilcoxon rank-sum test, LPS group: 20.1 ± 9.3 %; LPS + InPhase group: 16.8 ± 3.7 %; $P = 0.1183$
**11**	**Figures 5C-5E**	AntiPhase E-Stim did not improve behavior in OFT …	Wilcoxon rank-sum test, Time spent in the center of OFT : 3.7 ± 2.3 %, $P = 0.4550$, Figure 5C; Number of entries to center in OFT: 7.2 ± 5.8, $P = 0.4545$, Figure 5D; Distance travelled per unit of time in OFT: 7.9 ± 3.2 cm/s, $P = 0.0783$, Figure5E
**11**	**Figures 5F-5I**	… and EPM tests	Wilcoxon rank-sum test, Time spent in open arms of EPM : 12.3 ± 11.7 %, $P = 0.3410$, Figure 5F; Time spent in closed arms of EPM: 71.2 ± 17.8 %, $P = 0.2766$, Figure 5G; Time spent in center of EPM : 16.5 ± 11.9 %, $P = 0.1677$, Figure 5H; Distance travelled per unit of time in OFT: 3.6 ± 1.7 cm/s, $P = 0.2766$, Figure 5I

Table S2. Statistical table. *Related to STAR Methods - #Statistical analysis.*

Provided as a separate Excel file.

Table S3. Mouse CNO water daily consumption (g/day). *Related to STAR Methods - #Chemogenetic inhibition of OB neurons.*

Rat #	mCherry								hM4Di						
	**143**	**145**	**139**	**152**	**153**	**154**	**156**		**133**	**136**	**134**	**150**	**151**	**157**	**158**
**Day 1**	1.38	2.73	2.21	1.90	2.34	2.37	1.24		2.74	1.78	2.25	2.42	1.97	2.65	2.08
**Day 2**	2.90	3.26	3.09	2.47	2.98	2.68	2.52		3.50	2.41	3.80	2.78	2.81	2.37	2.54
**Day 3**	2.50	4.34	2.49	3.17	3.75	4.29	2.23		3.68	3.18	4.57	3.96	3.52	2.83	3.66
**Day 4**	2.61	4.06	2.96	2.77	3.20	3.05	2.04		3.62	3.29	3.55	3.17	3.83	3.02	3.50
**Day 5**	3.35	4.36	2.83	2.98	2.95	3.92	3.81		4.38	3.27	4.26	2.97	3.10	3.02	3.46
**Day 6**	3.74	4.15	3.63	2.79	3.58	3.49	3.95		5.01	2.53	3.84	3.23	3.82	3.58	3.60
**Day 7**	2.88	3.94	4.29	3.59	3.47	4.33	2.20		4.53	2.85	3.82	3.25	3.95	2.86	2.99
**Day 8**	2.20	3.71	2.99	3.26	3.58	3.15	3.43		3.56	3.39	3.64	3.12	3.55	3.05	3.45
**Day 9**	2.24	4.41	3.07	3.39	3.84	2.89	3.42		3.72	2.65	4.57	2.93	3.75	3.71	2.86
**Day 10**	3.09	4.01	3.70	4.02	3.83	3.71	3.79		3.38	2.96	4.06	3.74	4.02	4.48	3.45
**Day 11**	2.54	3.83	3.28	4.59	3.98	4.84	3.58		3.67	3.50	3.23	4.06	3.84	4.28	4.23
**Day 12**	4.14	3.65	2.84	2.87	3.24	3.60	3.59		3.54	2.44	3.95	3.46	3.33	3.34	4.60
**Day 13**	3.83	3.85	2.84	3.25	3.22	3.88	3.45		3.23	3.93	2.94	3.60	3.51	3.72	4.00
**Day 14**	4.33	3.93	2.65	2.97	3.33	4.33	4.00		4.48	3.25	3.25	3.62	3.43	4.34	4.22
**Day 15**	3.86	3.26	2.73	2.59	3.43	4.07	3.38		2.54	2.88	3.42	3.40	2.59	3.90	3.66
**Day 16**	4.33	3.95	3.88	3.46	3.20	3.81	4.00		3.19	3.69	3.90	3.84	3.36	4.67	3.85
**Day 17**	4.29	5.23	5.24	3.32	3.97	4.64	4.21		3.99	3.67	4.42	3.31	4.11	4.39	4.57
**Day 18**	5.07	5.37	5.17	3.39	3.76	4.27	3.62		2.58	3.02	4.08	3.85	3.56	4.00	4.68
**Day 19**	4.84	4.11	3.57	3.52	3.73	3.90	4.05		3.34	3.07	3.12	3.47	3.56	4.55	4.39
**Day 20**	3.87	4.93	3.66	3.55	2.59	4.18	3.19		2.83	3.39	3.48	3.02	2.72	4.67	4.06
**Day 21**	3.79	3.55	2.86	5.10	3.93	4.82	4.75		2.80	3.42	4.10	4.53	4.31	4.82	4.41
**Day 22**	4.18	4.62	3.14	3.21	3.43	4.23	2.22		2.39	3.28	3.10	3.12	2.96	5.18	3.77
**Day 23**	3.72	3.96	4.17	2.81	3.23	3.70	3.88		3.62	3.83	3.09	3.10	3.36	3.52	4.13
**Day 24**	3.24	3.23	1.93	2.89	3.15	4.57	3.24		2.79	3.37	2.99	4.62	3.18	3.54	3.97
**Day 25**	4.16	3.16	3.05	2.62	3.26	4.07	3.87		3.71	3.33	3.68	2.68	3.82	3.39	3.82
**Day 26**	3.86	3.22	3.60	3.30	3.50	4.08	3.61		2.93	3.18	3.60	4.08	4.05	3.59	3.46
**Day 27**	4.14	3.44	3.27	3.21	3.04	3.48	3.79		3.80	3.01	2.87	3.40	3.60	3.67	3.96
**Day 28**	3.74	3.69	3.13	3.56	3.69	4.15	4.31		2.77	2.75	2.58	3.75	3.76	3.38	3.25
**Day 29**	2.89	2.88	1.78	2.98	3.15	3.78	2.95		2.30	3.06	3.84	3.47	3.54	3.48	3.81
**Day 30**	4.50	4.60	3.97	3.15	3.32	4.15	3.54		2.19	3.79	4.81	3.35	3.48	3.73	3.74

Table S4. Electrodes implantation coordinates table. *Related to STAR Methods - #Electrode implantation surgery.*

BRAIN AREAS	AP	ML	DV/DISTANCE*	ANGLE
Brain-wide gamma oscillations in intact animals (**Figures S1 A–D**)				
Olfactory bulb (OB)	– 8.0 mm	+ 1.0 mm	1.4, 1.8 and 2.2 mm	N/A
Prelimbic cortex/infralimbic cortex (PrL/IL)	– 3.25 mm	+ 0.5 mm	2.0, 3.0 and 4.0 mm	N/A
Nucleus accumbens (NAc)	– 2.0 mm	+ 1.5 mm	6.5, 7.0 and 7.5 mm	N/A
Piriform cortex (PirC)	– 2.0 mm	+ 4.0 mm	6.5, 7.0 and 7.5 mm	N/A
Central amygdala/basolateral amygdala (CeA/BLA)	+ 2.2 mm	+ 3.0 and + 4.5 mm	7.5, 8.0 and 8.5 mm	6° from the parasagittal plane
Ventral tegmental area (VTA)	+ 5.3 mm	+ 1.0 mm	7.2, 7.6 and 8.0 mm	6° from the coronal plane
Ventral hippocampus (vHip)	+ 8.3 mm	+ 4.0 and + 5.0 mm	7.0, 7.5 and 8.0 mm	18° from the coronal plane
Brain-wide gamma oscillations in OBx animals (**Figures S1 E-H**)				
Secondary motor cortex (M2)	– 4.2 mm	+ 1.75 mm	1.0, 1.5 and 2.0 mm	N/A
Prelimbic cortex/infralimbic cortex (PrL/IL)	– 3.25 mm	+ 0.5 mm	2.0, 3.0 and 4.0 mm	N/A
Medial nucleus accumbens (midNAc)	– 2.0 mm	+ 1.0 mm	6.0, 6.5 and 7.0 mm	N/A
Lateral nucleus accumbens (latNAc)	– 2.0 mm	+ 2.5 mm	6.0, 6.5 and 7.0 mm	N/A
Piriform cortex (PirC)	– 2.0 mm	+ 4.0 mm	6.5, 7.0 and 7.5 mm	N/A
Anterior cingulate cortex (antCgC)	– 0.48 mm	+ 0.5 mm	1.0, 1.5 and 2.0 mm	N/A
Posterior cingulate cortex (postCgC)	+ 0.84 mm	+ 0.5 mm	1.0, 1.5 and 2.0 mm	N/A
Somatosensory cortex (S1)	+ 1.20 mm	+ 3.0 mm	1.0, 1.5 and 2.0 mm	N/A
Entopeduncular nucleus (EP)	+ 2.4 mm	+ 2.75 mm	7.0, 7.4 and 7.8 mm	N/A
Ventral posterolateral thalamic nucleus (VPL)	+ 2.4 mm	+ 3.25 mm	5.0, 5.5 and 6.0 mm	N/A
Chemogenetic inhibition of OB neurons (Mice) (**Figure 1E**)				
Olfactory bulb (OB)	– 4.8 mm	± 0.5 mm	1.4 mm	N/A
Piriform cortex (PirC)	– 1.78 mm	+ 2.0 mm	4 mm	N/A
Chemogenetic inhibition of OB neurons (Rats) (**Figure S2**)				
Olfactory bulb (OB)	– 8.0 mm	± 1.0 mm	1.4, 1.8 and 2.2 mm	N/A
Piriform cortex (PirC)	– 2.0 mm	± 2.6 mm	6.8, 7.1 and 7.4 mm	10° from the parasagittal plane

Optogenetic inhibition of the OB to PirC synaptic transmission (**Figure 2**)				
Olfactory bulb (OB)	– 8.0 mm	± 1.0 mm	1.4, 1.8 and 2.2 mm	N/A
Piriform cortex (PirC)	– 2.0 mm	± 3.3 mm	7.2 mm	5° from the parasagittal plane
Closed-loop OB gamma driven electrical stimulation of PirC (**Figures 3, 5, S6 and S8**)				
Olfactory bulb (OB)	– 8.0 mm	± 1.0 mm	1.4, 1.8 and 2.2 mm	N/A
Anterior piriform cortex (PirCA)	– 2.0 mm	± 2.6 mm	7.4 mm	10° from the parasagittal plane
Middle piriform cortex (PirCM)	0.0 mm	± 3.5 mm	8.3 mm	10° from the parasagittal plane
Posterior piriform cortex (PirCP)	+ 2.0 mm	± 4.0 mm	9.0 mm	10° from the parasagittal plane
lateral entorhinal cortex (LEC)	+ 6.0 mm	± 4.0 mm	7.8, 8.1 and 8.4 mm	20° from the parasagittal plane
Closed-loop OB gamma driven electrical stimulation of PirC (**Figures 4, S9 and S10**)				
Olfactory bulb (OB)	– 8.0 mm	± 1.0 mm	1.4, 1.8 and 2.2 mm	N/A
Anterior piriform cortex (PirCA)	– 2.0 mm	± 3.3 mm	7.2 mm	5° from the parasagittal plane
Middle piriform cortex (PirCM)	0.0 mm	± 3.5 mm	8.5 mm	10° from the parasagittal plane
Posterior piriform cortex (PirCP)	+ 2.0 mm	± 4.0 mm	9.0 mm	10° from the parasagittal plane
Cingulate cortex/Prelimbic cortex/Infralimbic cortex (Cg/PrL/IL)	– 3.25 mm	± 0.5 mm	1.2, 2.9 and 4.3 mm	N/A
Nucleus accumbens (NAc)	– 2.0 mm	– 1.5 or + 1.5 mm	6.7, 7.1 and 7.5 mm	N/A
Basolateral amygdala (BLA)	+ 2.8 mm	– 4.6 or + 4.6 mm	7.6, 7.8 and 8 mm	N/A
Ventral hippocampus (vHip)	+ 5.5 mm	– 4.5 or + 4.5 mm	6.4, 6.7 and 7 mm	N/A

‘–’ means anterior/left from the bregma/middle line;

‘+’ means posterior/right from the bregma/middle line;

‘±’ means both hemispheres from middle line;

‘*’ DV or distance from dura.

Table S5. Virus injection coordinates table. *Related to STAR Methods - #Chemogenetic inhibition of OB neurons and #Optogenetic inhibition of the OB to PirC synaptic transmission.*

INJECTED AREAS (VIRUS)	AP	ML	DV/DISTANCE*	ANGLE
Mouse hM4Di/mCherry injection (**Figure 1**)				
Olfactory bulb (AAV5-hSyn-mCherry/AAV5-hSyn-hM4Di-mCherry)	– 5.4 mm	± 0.5 mm	0.5, 1.0 and 1.5 mm	N/A
	– 4.8 mm	± 0.7 mm	0.6, 1.3 and 2.0 mm	N/A
	– 4.2 mm	± 0.7 mm	0.6, 1.4 and 2.2 mm	N/A
Rat hM4Di/mCherry injection (**Figure S2**)				
Olfactory bulb (AAV5-hSyn-mCherry/AAV5-hSyn-hM4Di-mCherry)	– 8 mm	± 0.6 mm	1.4, 2.5 and 3.5 mm	N/A
	– 8 mm	± 1.4 mm	0.9, 2.0 and 3.0 mm	N/A
	– 7 mm	± 0.8 mm	1.4, 2.5 and 3.5 mm	N/A
	– 7 mm	± 1.6 mm	1.3, 2.0 and 2.7 mm	N/A
	– 6.1 mm	± 0.6 mm	2.4, 3.1 and 3.8 mm	N/A
Rat anatomy pathway injection (**Figure S4**)				
Olfactory bulb (AAV5-EF1α-DIO-iC++-EYFP)	– 8 mm	± 0.6 mm	1.4, 2.5 and 3.5 mm	N/A
	– 8 mm	± 1.4 mm	0.9, 2.0 and 3.0 mm	N/A
	– 7 mm	± 0.8 mm	1.4, 2.5 and 3.5 mm	N/A
	– 7 mm	± 1.6 mm	1.3, 2.0 and 2.7 mm	N/A
	– 6.1 mm	± 0.6 mm	2.4, 3.1 and 3.8 mm	N/A
Piriform cortex (AAV2R-CAGGS-Cre-myc)	– 2.0 mm	± 3.3 mm	6.6, 6.9 and 7.2 mm	5° from the parasagittal plane
Rat optogenetics miniSOG injection (**Figure 2**)				
Olfactory bulb (AAVDJ-CAGGS-Flex-SYP1-miniSOG-T2A-mCherry)	– 8 mm	± 0.6 mm	1.4, 2.5 and 3.5 mm	N/A
	– 8 mm	± 1.4 mm	0.9, 2.0 and 3.0 mm	N/A
	– 7 mm	± 0.8 mm	1.4, 2.5 and 3.5 mm	N/A
	– 7 mm	± 1.6 mm	1.3, 2.0 and 2.7 mm	N/A
	– 6.1 mm	± 0.6 mm	2.4, 3.1 and 3.8 mm	N/A
Piriform cortex (AAV2R-CAGGS-Cre-myc)	– 2.0 mm	± 3.3 mm	6.6, 6.9 and 7.2 mm	5° from the parasagittal plane

'–' means anterior from the bregma;
'±' means both hemisphere from middle line;
'*' DV or distance from dura.
